# Energy driven constitutive modeling of thermally damaged red sandstone under multilevel cyclic loading

**DOI:** 10.1371/journal.pone.0357081

**Published:** 2026-11-04

**Authors:** You-Liang Chen, QingLong You, Jinbo Xie, Xi Du, Bo Lu, Pengjiao Jia, Tomas Manuel Fernandez-Steeger, Rafig Azzam

**Affiliations:** 1 Department of Civil Engineering, University of Shanghai for Science and Technology, Shanghai, China; 2 Department of Engineering Geology and Hydrogeology, RWTH Aachen University, Aachen, Germany; 3 School of Resources and Civil Engineering, Northeastern University, Shenyang, China; 4 Department of Engineering, University of Cambridge, Cambridge, United Kingdom; 5 School of Rail Transportation, Soochow University, Suzhou, China; 6 Department of Engineering Geology, Institute of Applied Geosciences, Technical University of Berlin, Berlin, Germany; The University of Hong Kong, HONG KONG

## Abstract

This study investigates the mechanical and energy responses of red sandstone subjected to high-temperature treatment and multilevel cyclic loading and develops an energy-driven constitutive model for its deformation behavior. Triaxial cyclic loading tests were conducted on red sandstone treated at different temperatures to examine the effects of thermal treatment and repeated loading on stress–strain response, residual deformation, and energy evolution. The results show that temperature significantly affects the mechanical properties and deformation characteristics of red sandstone. Under repeated loading at the same stress level, the hysteresis-loop area, residual-strain growth, and dissipated energy generally decrease with increasing cycle number, whereas an increase in stress level leads to renewed deformation and energy dissipation. Based on the experimental response, a staged constitutive framework is established for the initial loading, cyclic loading, and subsequent monotonic loading stages by considering compaction deformation, skeleton deformation, and damage evolution. Thermal and mechanical damage are incorporated through a load-carrying ratio, and energy density is introduced to characterize damage accumulation. The proposed model reproduces the main stress–strain characteristics observed in the tests and provides a constitutive description of the mechanical response of thermally treated red sandstone under multilevel cyclic loading.

## 1. Introduction

A central question is how to connect initial compaction, deformation and energy changes during stepped cyclic loading, and subsequent monotonic failure in one constitutive description of heat-treated red sandstone. Sandstone occurs in underground engineering, including the Yangcheng Tunnel of the Menghua Railway [1]. Heat released in nuclear-waste disposal can affect the surrounding rock [2,3]. In new types of underground gas storage (such as compressed air energy storage caverns), the rock layer must bear periodic injection–production loads together with temperature changes [4,5]. Studies show that coupling between groundwater and temperature can greatly change the sealing capacity and stability of the reservoir [6]. These applications motivate a macroscopic description of thermal-treatment and loading-history effects.

Previous studies associate thermal treatment with mineralogical and crack-related changes and altered rock strength and stiffness [7–10]. For example, high-temperature tests on limestone found that when the heating temperature is above 400°C, both peak stress and elastic modulus fall clearly, and the material becomes more brittle [11,12]. In the same way, higher temperature can increase the deformability of red sandstone. When the temperature is above 450°C, the stress–strain curve shows a local maximum before the main peak, meaning the peak stress appears earlier. This suggests that high temperature not only changes the static mechanical parameters of the rock, but also strongly affects its dynamic response [13,14]. Thermal-treatment studies also report changes in strength, stiffness and cracking [15–17].

In cyclic loading, rocks under repeated loading usually show typical features such as cyclic hardening or softening, hysteresis effects, and energy build-up. As the number of cycles increases, plastic deformation often becomes smaller, while the elastic modulus tends to rise. Under some conditions, the cyclic strength may even be higher than the strength from a single loading. At the same time, higher temperature can speed up crack growth and crack linking, making the failure pattern more complex [18–21]. These results suggest that multi-level cyclic loading can cause the internal damage of rock to change from fast growth to a more stable stage, and the material can show stronger elastic recovery in later cycles.

On the other hand, at the same stress level, more cycles can clearly change the shape of the hysteresis loops and the level of energy dissipation. In particular, when the rock is close to a fatigue instability threshold, the trends of dissipated energy, damage variables, and plastic strain may show clear turning points. This reflects the non-linear and stage-like nature of cyclic damage build-up [22,23]. In addition, when the cyclic stress reaches a critical level, the rock is more likely to show softening, and higher temperature often makes this softening stronger [24–26]. Overall, under multi-level cyclic loading, rock damage grows step by step and depends on the loading path. Stress level and cycle number together control the mechanical response. For the present stepped loading path, the relevant question is how energy evolution relates to deformation within a level and changes at level transitions.

In energy analysis and constitutive modeling, in recent years researchers have gradually combined the idea of energy conservation with damage theory, so they can explain rock behavior under high temperature and cyclic loading in a more reasonable way. A common view is that rock failure is essentially a process where energy changes from recoverable elastic stored energy to unrecoverable damage dissipation. So, using energy variables as the driving force of damage has strong physical meaning. Based on this idea, one type of model uses statistical damage or probability distributions to describe the scattered strength of micro-elements, and then combines a yield rule or an equivalent strain assumption to build thermo-mechanical coupled damage relations. Such models can, to some extent, show the stiffness drop and strength loss caused by temperature [27,28]. Another type of model puts more focus on bringing a thermodynamics framework into constitutive derivation, and uses energy dissipation or energy thresholds to describe damage start and damage growth, which improves the interpretability of the model [29].

The present study addresses the connection between these macroscopic stages under a single constant-confinement test path. Its contribution is the organization of a compaction description, a history-dependent cyclic formulation and two alternative post-cycling monotonic formulations within a common load-carrying framework. It does not propose a new general thermodynamic theory of rock damage.

Strain equivalence, a scalar modulus-based thermal damage indicator and Weibull statistical damage are adopted modeling concepts. The exponential compaction expression is adapted from the pore/crack closure relation discussed in Section 4.1. Their use in the staged framework is distinguished here from the experimentally observed stress–strain and energy trends.

Table 1 places the proposed framework alongside existing damage models and summarizes the differences in scope and parameter requirements.

**Table 1:** Comparison of different damage models for rock cyclic fatigue.

	Core idea	Scope	Comments
[30]	Uses irreversible deformation to characterize fatigue damage, establishes an inverted S-shaped nonlinear cumulative damage law, and introduces a generalized cycle number/fatigue path to address cumulative effects under complex loading.	Rock cyclic fatigue under constant-amplitude, multi-level, and random-spectrum loading.	Simple and calibratable, and can partly capture loading-sequence and cumulative effects; however, it remains relatively empirical and usually requires re-calibration for different rock types or loading paths.
[31]	Builds a damage constitutive model based on strain equivalence, introduces a compaction factor for the initial compaction stage, and reconstructs cyclic loading–unloading curves recursively to reproduce hysteresis and stiffness degradation.	Reconstruction and fitting of cyclic loading–unloading curves for rocks/soft rocks with a pronounced compaction stage.	Effectively describes compaction nonlinearity and hysteresis, and is convenient for engineering implementation; however, it is mainly oriented to curve reconstruction, while the mechanism-level damage-state updating framework remains relatively weak.
[32]	For jointed rock masses, couples micro-crack statistical damage with macro-joint effects, introduces internal variables/internal time, and uses irreversible strain to describe hysteresis and residual deformation, with correction for the first-loading compaction stage.	Uniaxial cyclic loading–unloading of discontinuous jointed rocks, including stiffness/strength degradation, residual strain, and hysteresis.	Can jointly capture hysteresis, residual strain, and degradation, with more direct representation of joint effects; however, the model involves many parameters, high calibration cost, and limited proof of transferability across joint morphologies and thermal-structure differences.
[33]	Defines damage using normalized deformation and adopts a two-term power function to describe initial, stable, and accelerated damage growth, with empirical relations between parameters and loading conditions.	Salt rock fatigue under different stress amplitudes, frequencies, and loading rates.	The three-stage evolution matches experiments well and supports life estimation; however, it depends strongly on the fitted data range and regression form, so generalization is limited.
[34]	Establishes a cyclic constitutive model based on state variables, where state/hardening evolution reflects loading history and crack-related damage measures are used to update the damage state.	Salt rock cyclic loading–unloading and path-dependent response.	Shows good predictive accuracy under different cyclic paths and can better reflect deformation–stress-path interaction; however, the initial loading stage still requires additional correction for compaction and time-independent plasticity, and parameter calibration demand remains high.
[35]	Defines damage through dissipated-energy ratio and proposes a stage-based damage evolution law to explain cyclic damage accumulation.	Rock cyclic fatigue, such as sandstone, for damage-evolution description.	The damage variable has clear physical meaning and the stage-based form matches experiments well; however, the definitions of total dissipated energy at failure and damage-effective dissipated energy remain difficult, and broader applicability still requires further validation.

Red sandstone treated at four temperatures is examined using three cyclic stress levels followed by monotonic loading to failure. The analysis focuses on the response within each level, changes at stress-level switching and reproduction of the measured curves by the staged model. Generalization to other loading paths requires further assessment.

## 2. Test program

### 2.1. Specimen preparation

This study used cylindrical red sandstone specimens. Each specimen measured 50 mm × 50 mm × 100 mm. The rock samples were generally non-bedded, to reduce the effect of anisotropy on the mechanical response as much as possible. One valid specimen with a complete test record was used for each temperature condition (25, 300, 600, and 900 °C). The reported curves are the actual individual responses, not averages or representative curves selected from complete replicate tests. No valid replicate dataset was available for calculating standard deviations, error bars or repeatability statistics.

All specimens were pre-dried at 105 °C for 6 h to reduce differences in initial moisture content. The 25 °C reference specimen was cooled to room temperature after this same pre-drying treatment and then mechanically tested. The high-temperature specimens were heated at 5 °C/min to 300, 600 or 900 °C, held at the target temperature for 2 h, and naturally cooled in the furnace to room temperature before mechanical testing. The heating rate was selected to minimize the potential influence of thermal shock while maintaining reasonable experimental efficiency. No separate tests were performed to quantify the respective damage contributions of heating, holding and cooling, so the results reflect the combined thermal-treatment effects. The heating curve is shown in Fig 1.

**Fig 1:**
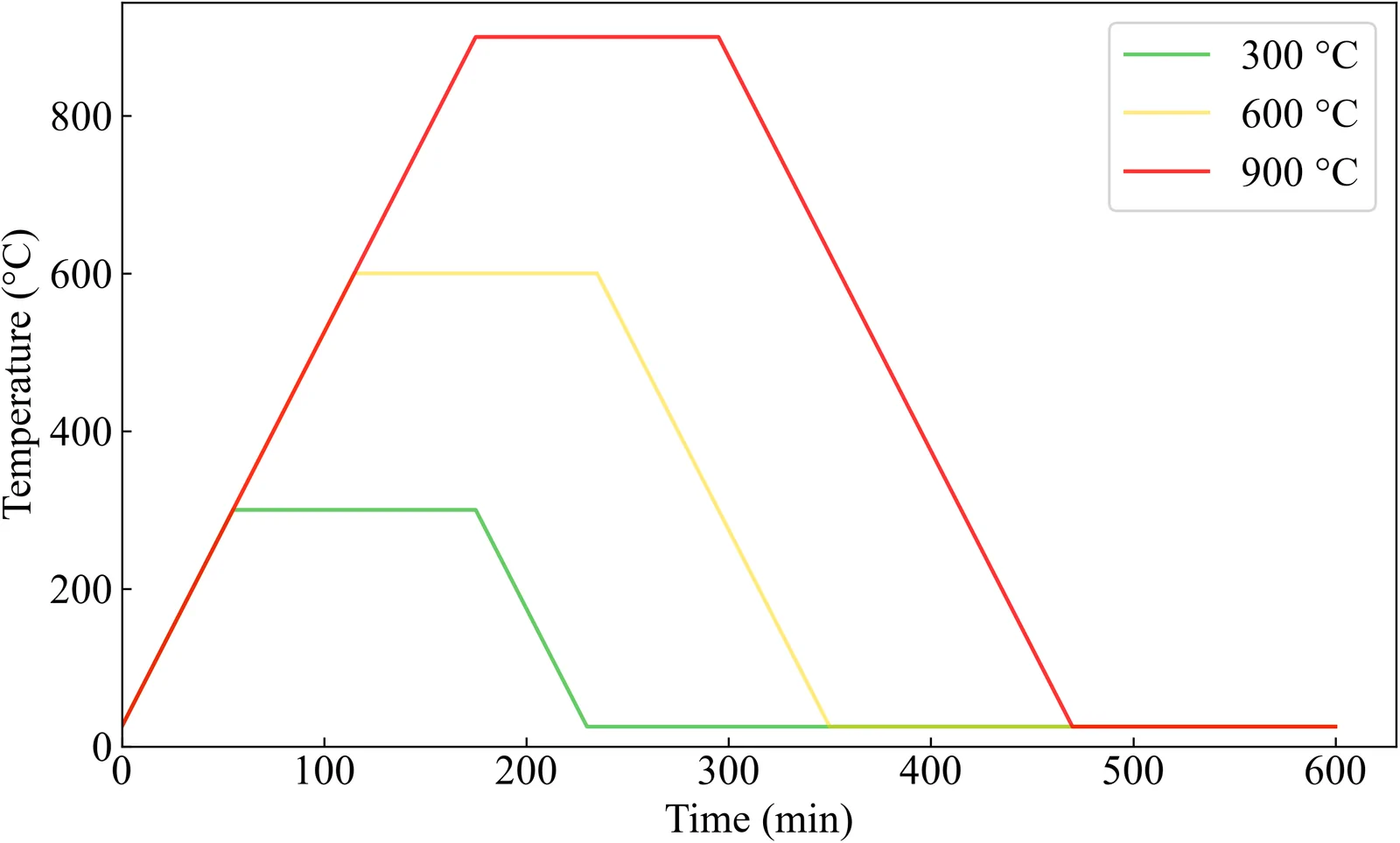
Heating curve.

In the triaxial loading stage, axial loading was applied under stress control (see the device sketch in Fig 2). The loading path is shown in Fig 3. This setup helps keep the multi-level cyclic loading–unloading path stable and well controlled, and provides consistent test conditions for later stress–strain and energy-evolution analysis (see Fig 2 for the triaxial compression device sketch).

**Fig 2:**
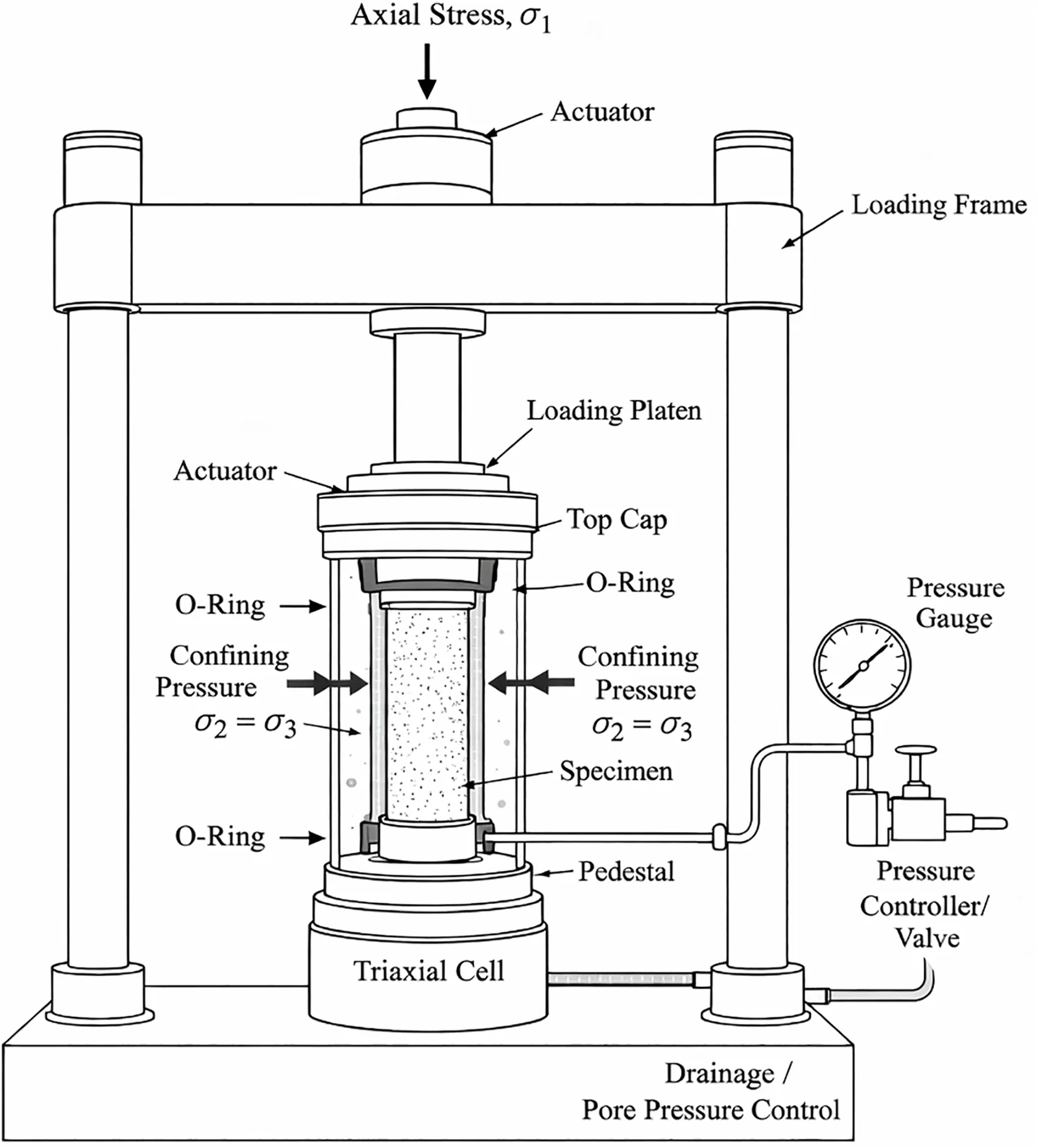
Sketch of the triaxial compression device ($\sigma_{2}=\sigma_{3}$).

**Fig 3:**
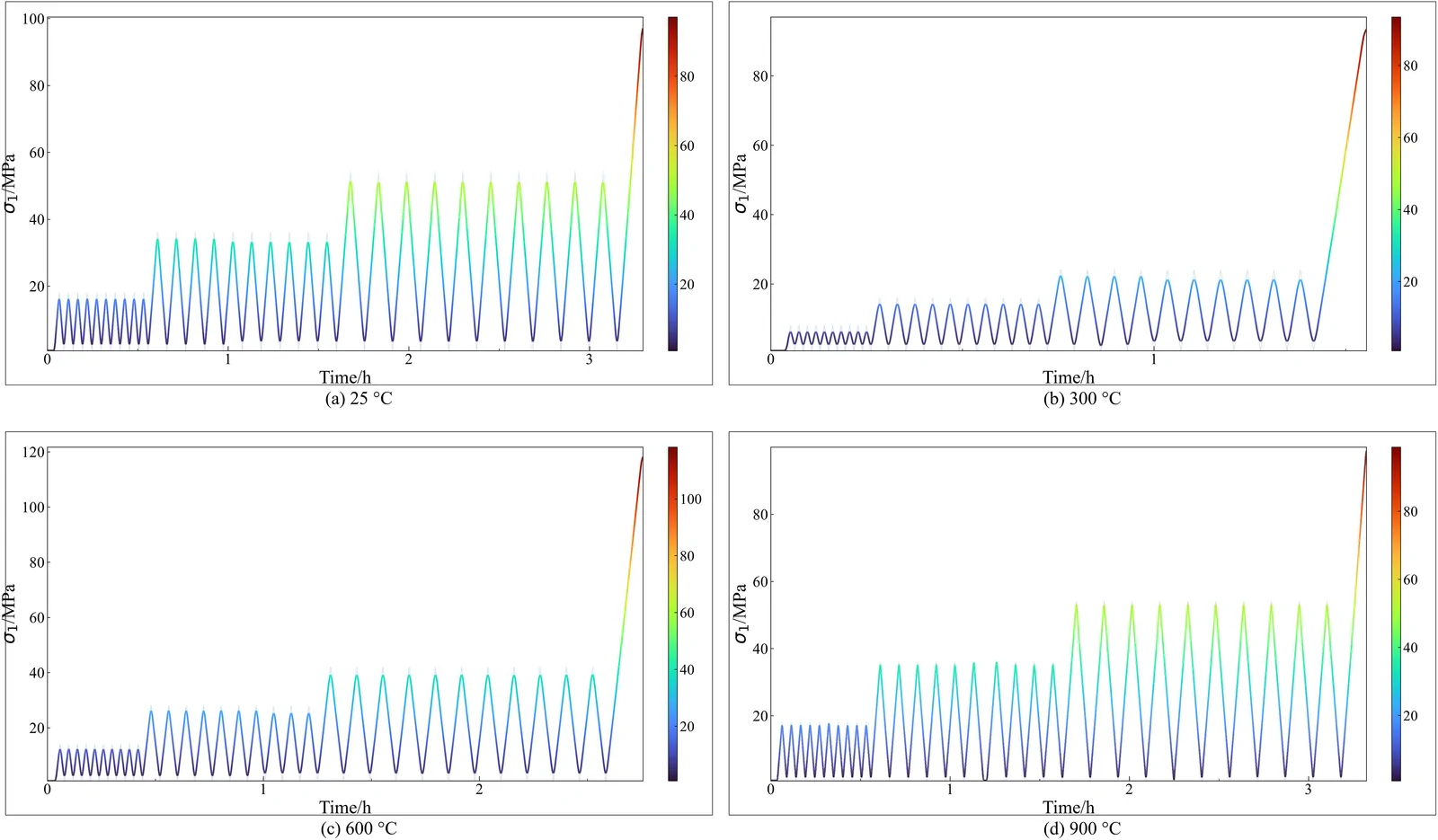
Loading path of the major principal stress. (a) 25°C. (b) 300°C. (c) 600°C. (d) 900°C.

### 2.2. Multi-level cyclic loading–unloading and final crushing test under constant confining pressure

The mechanical tests were carried out under triaxial conditions, and the confining pressure was kept constant at $\sigma_{\text{3}}=\text{1}\text{0}\;\text{MPa}$ during the whole loading history. Axial loading followed a path of multi-level cyclic loading–unloading, and then final monotonic compression to failure. Three axial stress levels were set, named Level I, Level II, and Level III, and they satisfy $\sigma_{\text{III}}>\sigma_{\text{II}}>\sigma_{\text{I}}.$ Table 2 lists the total axial stress upper limits, measured peak strengths and descriptive stress-to-peak ratios. The programmed loading and unloading rates were both set to 0.2 MPa/s. The nominal lower axial stress limit was 0 MPa, with a small positive load retained by the apparatus to maintain contact. The different absolute upper limits accommodated the load-bearing capacity after each thermal treatment while allowing completion of all three cyclic levels and subsequent monotonic loading to failure. Each condition retained a 1:2:3 sequence; the levels were not prescribed as fixed fractions of peak strength and are not proposed as a universal loading scheme.

**Table 2:** Cyclic stress levels and their ratios to the measured peak strength.

Temprature (°C)	Level I (MPa)	Level II (MPa)	Level III (MPa)	Measured peak (MPa)
25 °C	18	36	54	100.4592
300 °C	8	16	24	96.9600
600 °C	14	28	42	121.7028
900 °C	20	40	60	127.9440

Stress levels and peak strength are total axial stresses.

First, the specimen was loaded to the prescribed upper stress of Level I and subjected to cyclic loading–unloading between the nominal lower and upper stress limits. The same procedure was subsequently applied at Levels II and III, after which the specimen was loaded monotonically to failure. The nominal loading protocol specified 10 cycles at each stress level. In the valid 600 °C test record, however, 11 cycles were completed at Level III, and the corresponding experimental results were analyzed according to the actual recorded loading history. The zero lower bound specified in the loading protocol represents the nominal control limit, whereas the measured minimum stress during testing remained slightly positive.

After the Level III cycles were finished, the confining pressure $\sigma_{\text{3}}$ was still kept unchanged, and the specimen was then loaded by axial monotonic compression until failure, to obtain the peak strength $\sigma_{\text{peak}}$ and the response up to peak failure. During the test, raw data such as axial stress $\sigma_{\text{1}}$ and axial strain $\varepsilon_{\text{1}}$ were recorded at the same time. A typical curve of $\sigma_{\text{1}}$ versus time is also provided (the major principal stress loading path is shown in Fig 3) to show the multi-level cyclic loading program and the stability of the control. Peak stress was defined as the maximum measured axial stress at failure. Quantitative analysis was restricted to the record up to this peak. After peak failure, the specimen fractured and the system unloaded rapidly; the sparse post-peak measurements were insufficient to characterize a reliable complete post-peak response. The experimental curves use the raw measurements without interpolation, resampling, filtering or smoothing. Residual strain is the measured axial strain at the minimum-stress endpoint of each unloading branch.

## 3. Experimental results and analysis

### 3.1. Stress–strain response and multi-level cyclic features

Fig 4 presents the measured stress–strain histories across stress-level transitions, cycling within each level and final monotonic loading.

**Fig 4:**
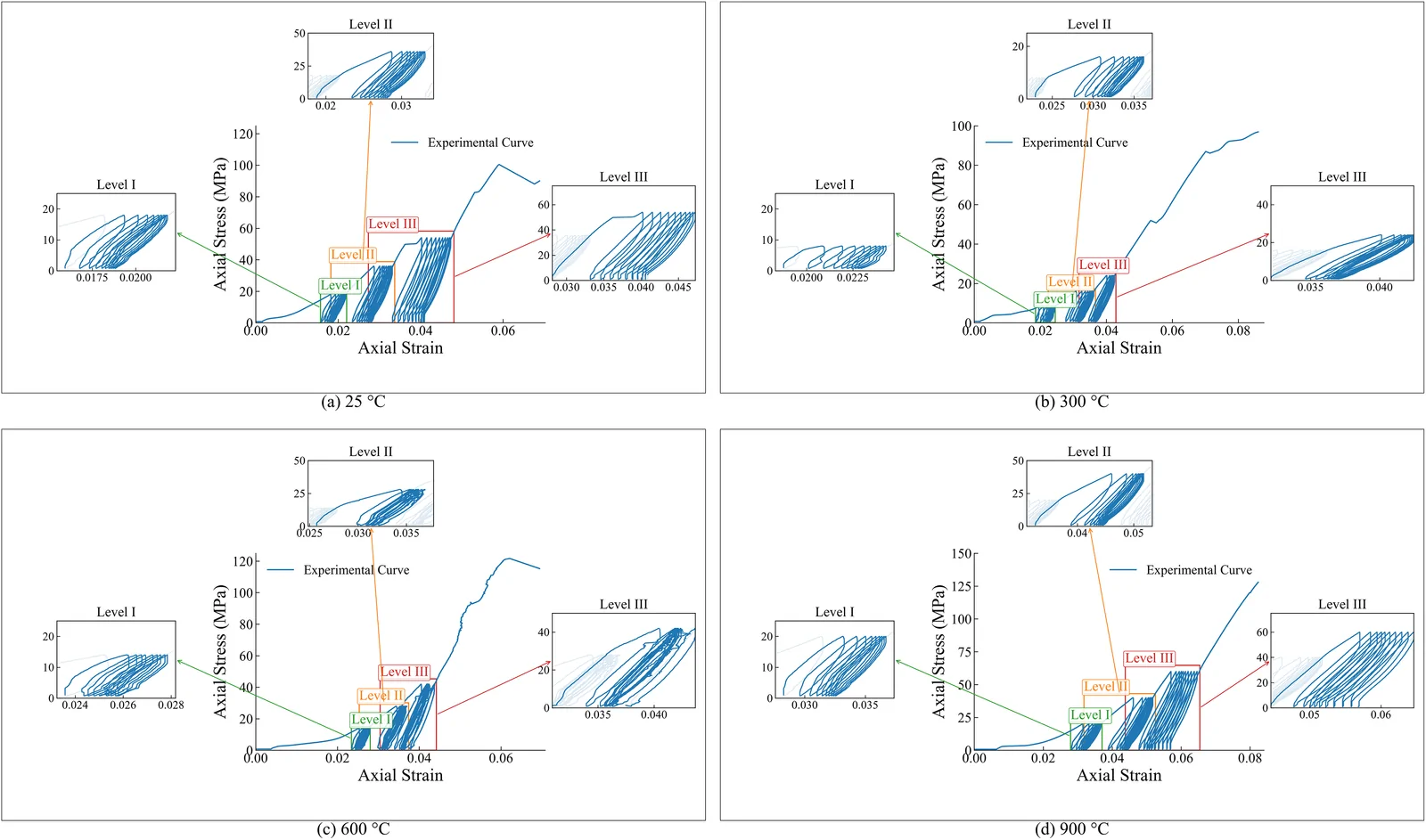
Stress–strain curves along the time series. (a) 25°C. (b) 300°C. (c) 600°C. (d) 900°C.

From the overall response at different stress levels, as the stress level rises from Level I to Level II and then to Level III, the linear feature of the stress–strain relation becomes stronger. The curve shows a clearer “near-linear loading segment”, and the corresponding equivalent stiffness also increases. For the cyclic response within the same stress level, as the number of cycles increases, the hysteresis loop area gradually becomes smaller, and the loading and unloading branches gradually move closer. This indicates that the increment of irreversible deformation keeps decreasing during cycling. At the same time, the increment of residual strain after unloading becomes smaller, showing that the added residual deformation caused by further cycling at the same stress level tends to be limited. Each time the loading changes from $\sigma_{\text{I}}$ to $\sigma_{\text{II}}$, or from $\sigma_{\text{II}}$ to $\sigma_{\text{III}}$, the hysteresis loop becomes larger again, and this is especially clear in the first few cycles after entering the new stress level. Then, as cycling continues at that level, the hysteresis loop becomes smaller again and tends to become stable. It should be noted that during the transition loading segment of stress-level switching, axial strain often shows a jump-like increase.

One possible interpretation of the within-level reduction in hysteresis is progressive closure or stabilization of compliant defects. Increased stress amplitude may then activate additional irreversible deformation.

### 3.2. Evolution of energy density and cyclic dissipation characteristics

Energy density was calculated from the raw measured total axial stress–axial strain data by u=∫σdε. Here the stress and strain denote the measured total axial stress and axial strain. For each cycle, input energy is the integral along the loading branch from its measured minimum-stress point to its maximum-stress point. Dissipated energy is the area of the complete loading–unloading hysteresis loop, and recoverable (elastic) energy is input energy minus dissipated energy. The measured small positive minimum stress is retained without forcing it to zero. The residual-strain endpoint is taken directly from the unloading record. Hysteresis loss represents irreversible dissipation and is not assigned entirely to damage. The energy results are shown from two views: (1) the full multi-level process, which describes the energy evolution from Level I to Level III and then to the final crushing stage; and (2) the level-by-level cycles, which compares the energy difference between the first cycle and the stable cycle within the same level, and its decay process. The energy-density results are shown in Figs 5–7.

**Fig 5:**
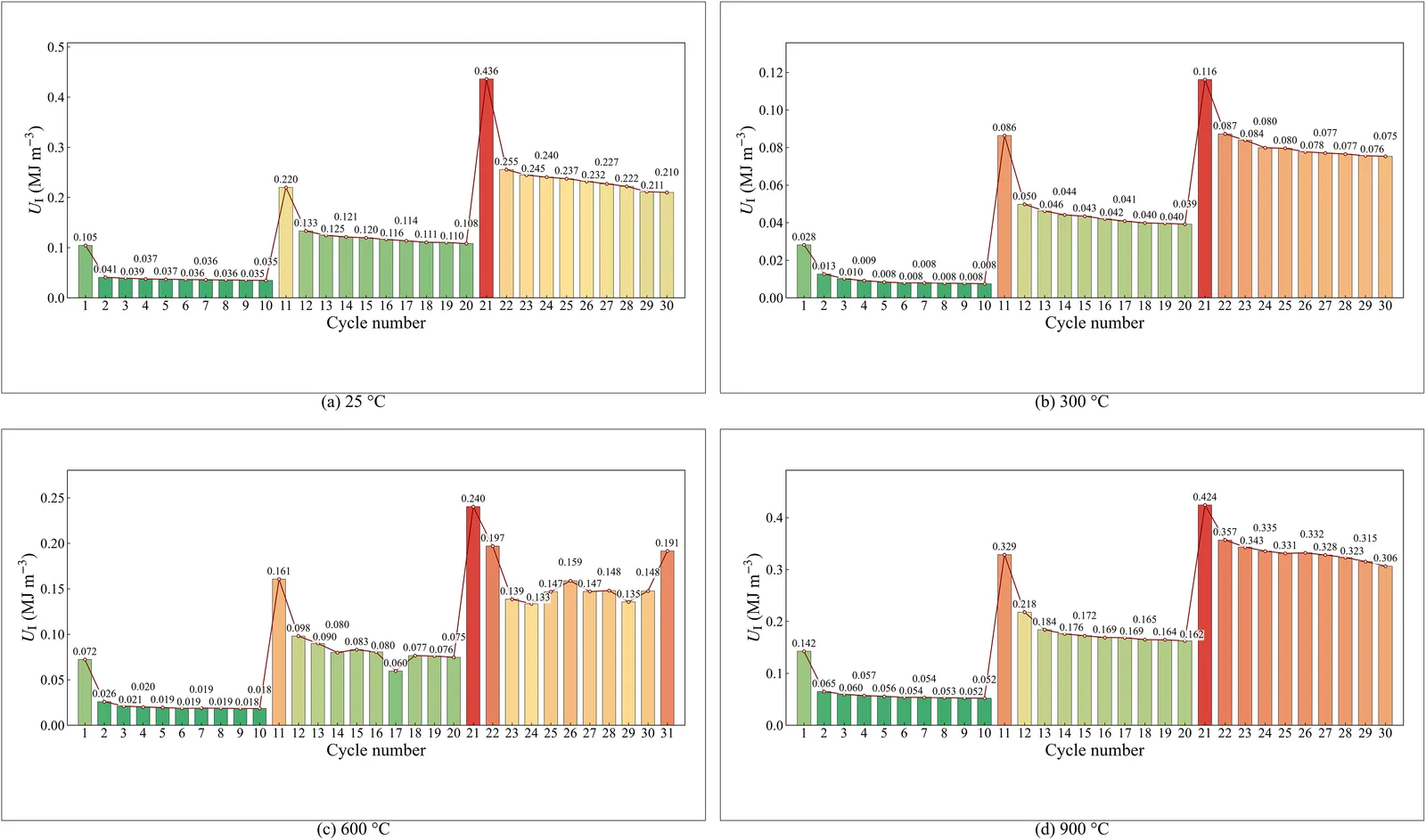
Total input energy density.

**Fig 6:**
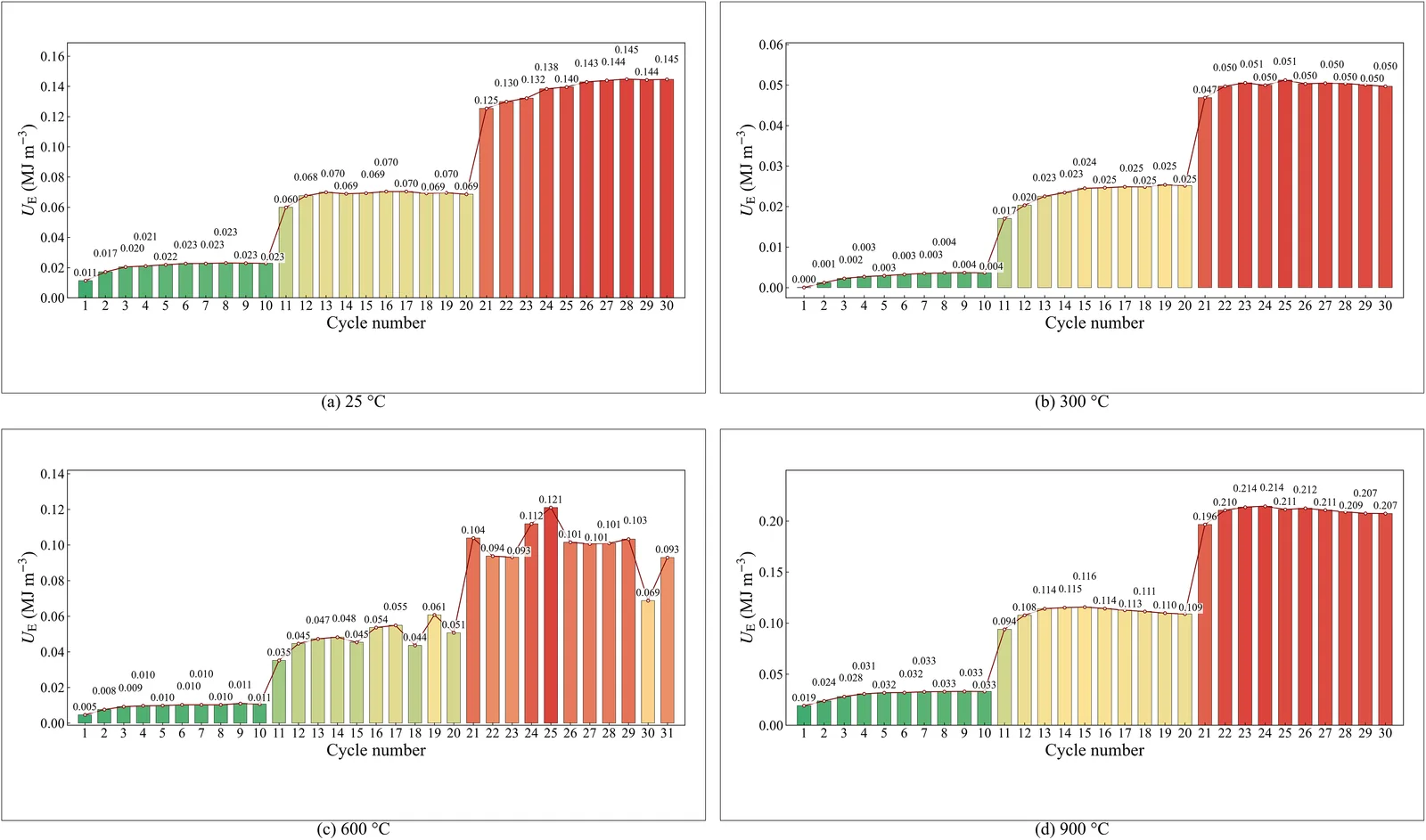
Elastic energy density curve.

**Fig 7:**
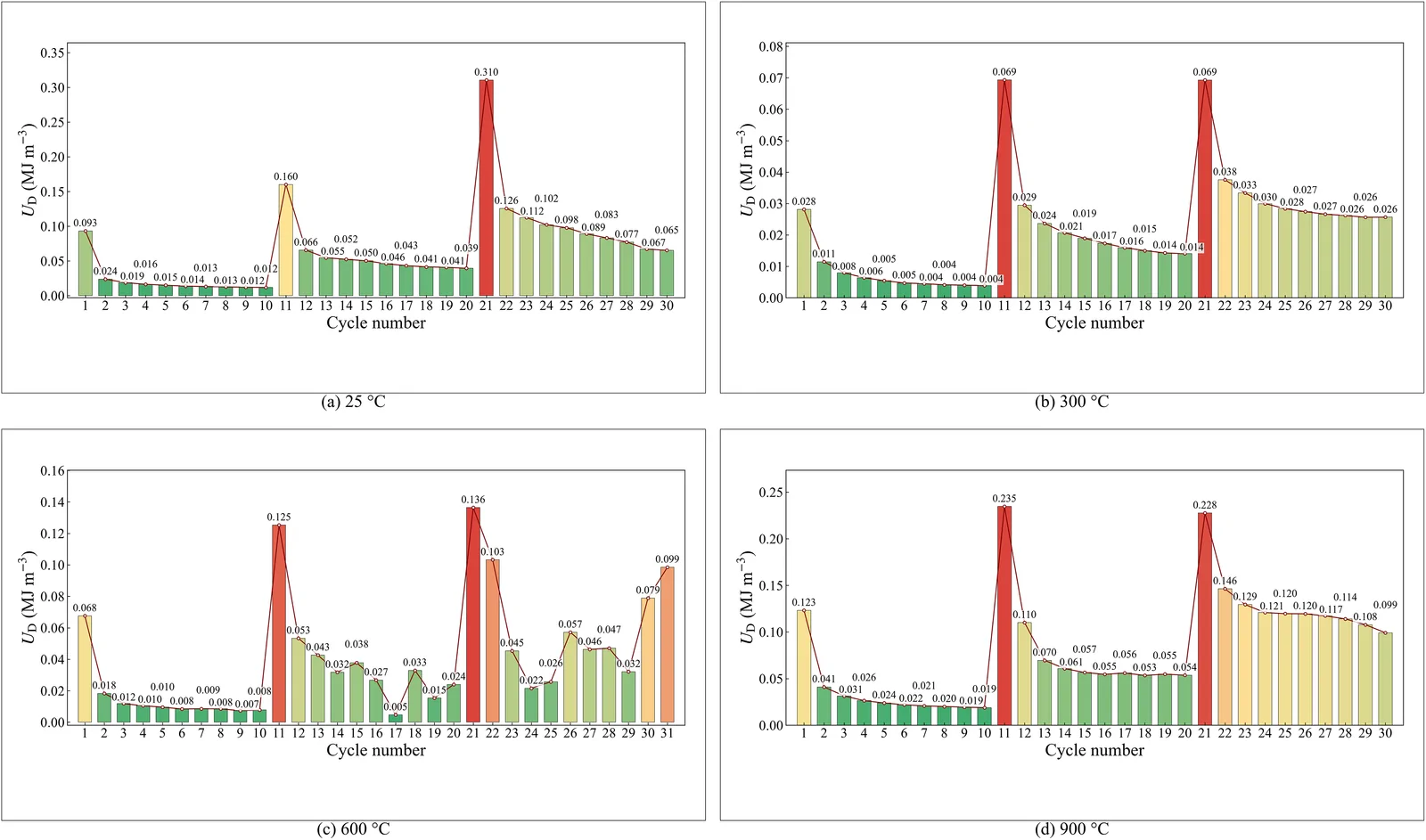
Dissipated energy density.

Across different stress levels, the total input energy density increases as the stress level increases. When loading jumps from a lower stress level to a higher one, because the stress amplitude rises and the strain shows a clear increase in the transition segment, the energy density often shows a sudden jump. By comparing the energy parts, we can see that this jump is mainly in the dissipated energy density. That is, during stress-level switching and in the early cycles at the new level, the hysteresis loop area becomes much larger, and the dissipated energy shows a peak-like feature. In contrast, during cycling within the same level, as the number of cycles increases, the total input energy density shows a gradual decrease. This decrease mainly comes from smaller deformation at the same stress level, which reduces the area of ∫σdε.

From the view of energy share, within the same stress level, as cycling continues, the elastic energy share in the input energy gradually increases, and shows a trend of increasing and then becoming stable. In contrast, the dissipated energy density gradually decreases with cycle number, and the decay is more monotonic and clear. This means that at the same stress level, loading–unloading in later cycles becomes closer to a recoverable elastic response, while the energy cost linked to irreversible processes keeps dropping. Since the stress upper limit in the same level is almost unchanged, the energy change should be understood mainly from “gradual convergence of deformation response”. In other words, cycling keeps weakening the added deformation and added irreversible processes within each cycle. This matches the stress–strain results where hysteresis loops shrink and residual-strain increments become smaller.

### 3.3. Stress–Strain interpretation from an energy view

The stress–strain and energy curves show a repeated sequence of declining dissipation within a level, renewed dissipation after a stress-level increase, and subsequent decline. The narrowing loops and smaller increments of residual deformation are consistent with a progressively more recoverable response over the observed cycles. Pore closure or contact stabilization may contribute, but these mechanisms are not directly resolved by the macroscopic curves.

When the stress level rises, the early cycles at the new level show wider loops and larger strain increments, together with increased dissipated energy. This links the measured deformation and energy trends without assigning the additional dissipation to a uniquely identified microscopic process.

## 4. Constitutive modeling under multi-stage loading

### 4.1. Stage I: Initial loading

Under conventional triaxial compression, the axial principal stress is $\sigma_{\text{1}}$, and the lateral principal stresses are equal to the confining pressure, i.e., $\sigma_{\text{2}}=\sigma_{\text{3}}$. In the small-strain range, if the material can be treated as an isotropic linear elastic body, the axial strain $\varepsilon_{\text{1}}$and the three principal stresses follow Hooke’s law $\varepsilon_{\text{1}}=\frac{\text{1}}{E}\left(\sigma_{\text{1}}-\nu (\sigma_{\text{2}}+\sigma_{\text{3}})\right).$Since $\sigma_{\text{2}}=\sigma_{\text{3}}$, we have $\varepsilon_{\text{1}}=\frac{\text{1}}{E}\left(\sigma_{\text{1}}-\text{2}\nu \sigma_{\text{3}}\right).$ Therefore, the effect of the confining pressure $\sigma_{\text{3}}$ on the axial response can be treated as a constant offset term $\text{2}\nu \sigma_{\text{3}}$in the axial stress. To build the later nonlinear mechanisms (such as compaction closure and damage growth) on the same “load-bearing part that drives axial deformation”, an equivalent stress $\sigma_{e}$ is introduced to separate the confining-pressure effect. We first model using $\sigma_{e}$, and then recover $\sigma_{\text{1}}$ at the end. The formulation assumes a small-strain, isotropic elastic skeleton, strain equivalence, separation of initial compaction from skeleton strain, and scalar thermal and mechanical damage. The product coupling treats mechanical degradation as acting on the remaining thermally degraded load-carrying fraction, without an explicit interaction variable. Stage I represents the initial compaction and loading response, Stage II represents unloading–reloading history, and Stage III represents monotonic loading from the inherited cyclic state. These are macroscopic modeling assumptions for the present constant-confinement tests; arbitrary stress paths, anisotropic damage and long-term rate effects are outside the demonstrated scope.


$$\begin{array}{c} \sigma_{e}=\sigma_{\text{1}}-\text{2}\nu \sigma_{\text{3}} \end{array}$$ (1)

$$\begin{array}{c} \sigma_{\text{1}}=\sigma_{e}+\text{2}\nu \sigma_{\text{3}} \end{array}$$ (2)

where $\sigma_{\text{1}}$ is the total axial stress, $\sigma_{\text{3}}$ is the confining pressure, and ν is Poisson’s ratio. This transformation represents the lateral Poisson contribution as an axial stress offset for fixed confinement and an assumed constant Poisson ratio. The model was calibrated and evaluated using the same experimental dataset, and no independent test dataset was available for validation. Therefore, the present comparison demonstrates the model’s ability to reproduce the fitted experimental responses rather than its independent predictive capability, which requires further verification using additional experimental data.

Heat-treated red sandstone usually shows clear nonlinear compaction at low stress. This mainly comes from the gradual closure of initial pores and pre-existing micro-cracks under compression. To separate “pore/crack closure” from “effective load-bearing deformation of the solid skeleton”, and to avoid mixing compaction nonlinearity with later damage growth, the total axial strain $\varepsilon_{\text{1}}$ is decomposed into two parts: the skeleton strain $\varepsilon_{s}$ and the extra compaction strain $\varepsilon_{v}$ caused by pore closure (see Fig 8):


$$\begin{array}{c} \varepsilon_{\text{1}}=\varepsilon_{s}+\varepsilon_{v} \end{array}$$ (3)

**Fig 8:**
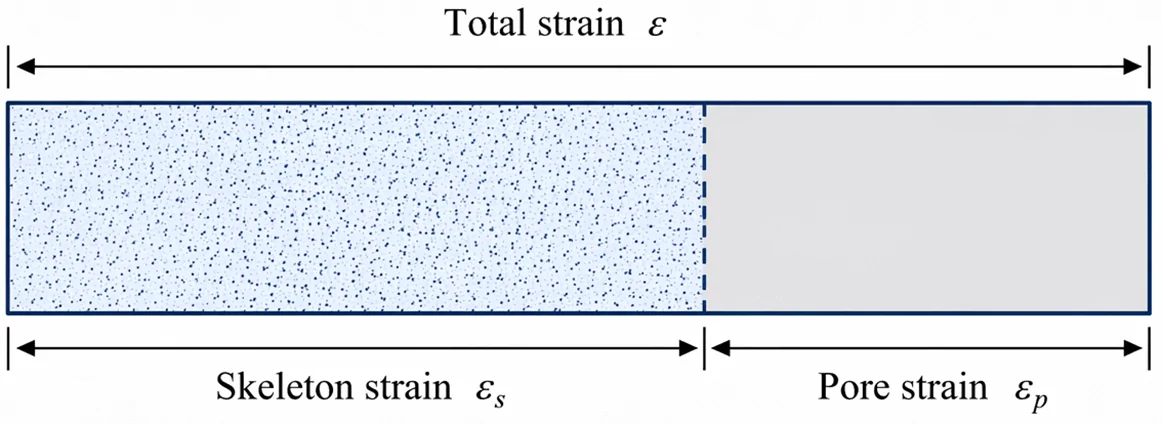
Schematic diagram of rock deformation components.

where $\varepsilon_{\text{1}}$ is the total axial strain; $\varepsilon_{s}$ is the strain of the effective skeleton deformation (the basic measure of recoverable/irrecoverable deformation of the solid skeleton under load); and $\varepsilon_{v}$ is the extra compaction strain caused by the gradual closure of pores and cracks.

With constant confining pressure and using the equivalent stress $\sigma_{e}$ (Eq. (1)) to represent the axial load-bearing part, pore/crack closure shows a nonlinear pattern of “fast first, then slow, and gradually saturating”. Referring to the exponential volume–stress relation derived by [36] for pore/crack volume in a “soft part”, $V_{t}=V_{\text{0},t}\text{exp}(-\sigma_{e}/\sigma_{c})$, this paper uses an exponential saturation function to describe the evolution of the closure (compaction) strain:


$$\begin{array}{c} \varepsilon_{v}(\sigma_{e})=\varepsilon_{v}^{c}[\text{1}-exp(-\sigma_{e}/\sigma_{c})] \end{array}$$ (4)

where $\varepsilon_{v}^{c}$ is the maximum closable compaction strain (the saturation value, describing the total amount of initial pores/cracks that can be compacted), and $\sigma_{c}$ is the characteristic closure-stress scale in the exponential compaction model (controlling the closure rate; a larger $\sigma_{c}$ means a higher stress is needed to reach the same closure level). The characteristic closure scale is distinct from the crack-closure threshold defined in Eqs. (8)–(11). In Eq. (4), the scale controls how rapidly compaction approaches its limiting value; the threshold identifies a selected point on the measured response. The equation does not require these two stresses to be equal. Temperature-specific parameters were obtained by constrained/regularized nonlinear least-squares calibration of Eq. (4) against the initial nonlinear compaction segments; these are not four fully unconstrained independent fits. Table 3 reports the parameters and stress-domain fit quality. The saturation strain increases from 25 to 600 °C and decreases at 900 °C, while the characteristic stress scale is largest at 300 °C; neither parameter follows a monotonic temperature trend across all four specimens.

**Table 3:** Stage I compaction parameters and stress-domain fit quality.

Temperature	$\varepsilon_{v}^{c}$	$\sigma_{c}$ (MPa)
25 °C	0.01094	6.211
300 °C	0.01951	10.700
600 °C	0.02162	6.209
900 °C	0.01993	4.755

Substituting Eq. (4) into Eq. (3) gives an explicit expression of the skeleton strain (i.e., removing the pore-closure part from the total strain):


$$\begin{array}{c} \varepsilon_{s}=\varepsilon_{\text{1}}-\varepsilon_{v}(\sigma_{e})=\varepsilon_{\text{1}}-\varepsilon_{v}^{c}[\text{1}-exp(-\frac{\sigma_{e}}{\sigma_{c}})] \end{array}$$ (5)

Eq. (5) plays a key role in the later derivation: any damage variable related to strength degradation (either energy-driven damage or statistical damage) should use $\varepsilon_{s}$ as the driving measure of skeleton deformation; while $\varepsilon_{v}$ only reflects the nonlinear compaction effect caused by pore-structure closure. In this way, compaction nonlinearity and damage nonlinearity remain separable in the math form, and stage division and parameter identification become more practical.

To reflect the effect of heat treatment on the initial stiffness, a thermal damage variable $D_{T}$ is introduced and defined by the reduction of elastic modulus:


$$\begin{array}{c} D_{T}=\text{1}-\frac{E_{T}}{E_{\text{0}}} \end{array}$$ (6)

Thus, the elastic modulus after heat treatment is


$$\begin{array}{c} E_{T}=(\text{1}-D_{T})E_{\text{0}} \end{array}$$ (7)

where $E_{\text{0}}$ is the reference elastic modulus of the pre-dried 25 °C reference specimen, $E_{T}$ is the elastic modulus of the specimen after heat treatment at the same temperature condition, and $D_{T}$ is the thermal damage variable. This scalar indicator summarizes the modulus change after the thermal-treatment history. It does not distinguish damage associated with heating, holding or cooling, and it does not identify a specific mineralogical mechanism.

The experimentally defined axial crack-closure threshold, $\sigma_{cc}$, is distinct from the fitted characteristic closure stress, $\sigma_{c}$, used in the exponential compaction relation. In principle, $\sigma_{cc}$ and the corresponding crack-closure strain, $\sigma_{cc}$, can be identified from the initial triaxial loading segment at each temperature by adapting the ASD (axial stress difference) method [37], without introducing additional uniaxial test data [38]. However, reliable independent ASD estimates could not be obtained from the present experimental records. Therefore, numerical values of $\sigma_{cc}$ and $\sigma_{cc}$ are not reported in this study.


$$\begin{array}{c} \left(\sigma_{cc},\varepsilon_{cc}\right) \end{array}$$ (8)

Converted into the equivalent-stress space:


$$\begin{array}{c} \sigma_{e,cc}=\sigma_{cc}-\text{2}\nu \sigma_{\text{3}} \end{array}$$ (9)

The compaction strain at the closure point is given by Eq. (4):


$$\begin{array}{c} \varepsilon_{v,cc}=\varepsilon_{v}(\sigma_{e,cc})=\varepsilon_{v}^{c}[\text{1}-exp(-\frac{\sigma_{e,cc}}{\sigma_{c}})] \end{array}$$ (10)

The skeleton strain at the closure threshold is defined as:


$$\begin{array}{c} \varepsilon_{s,cc}=\varepsilon_{cc}-\varepsilon_{v,cc} \end{array}$$ (11)

where $\sigma_{cc}$ and $\varepsilon_{cc}$ are the stress and strain at the crack-closure point to be identified from tests; $\sigma_{e,cc}$ is the corresponding equivalent stress; $\varepsilon_{v,cc}$ is the compaction strain at closure; and $\varepsilon_{s,cc}$ is the skeleton strain at closure.

Using whether $\sigma_{e}$ exceeds the crack-closure threshold $\sigma_{e,cc}$ as the criterion, Stage I is divided into two cases:


$$\begin{array}{c} \sigma_{e}\leq \sigma_{e,cc}\Rightarrow ``\text{stress only causes crack closure},\text{ and thermal damage exists}" \end{array}$$ (12)

$$\begin{array}{c} \sigma_{e}>\sigma_{e,cc}\Rightarrow after\;closure,\;the\;rock-grain\;skeleton\;develops\;stress\;damage \end{array}$$ (13)

According to the relationship between the effective stress $\sigma_{e}$ and the crack-closure threshold stress $\sigma_{e,cc}$, two cases are considered for the evolution of skeleton damage in Stage I. This is a conditional threshold within Stage I. If the end stress of Stage I remains below the equivalent closure threshold, the response stays in Case A and Case B is not activated before cycling begins. Reaching the threshold gives the continuous zero-mechanical-damage boundary between the cases, with Case B damage developing above it. In Stage II, the closure threshold is not reapplied as a switching criterion for each cycle.

Case A: when $\sigma_{e}\leq \sigma_{e,cc}$, the stress damage of the rock-grain skeleton is assumed to be zero. In this range, the stress mainly causes pore/crack closure. So cracks are still dominated by closure, and the skeleton bearing behavior is only affected by thermal damage:


$$\begin{array}{c} \sigma_{e}=E_{T}\varepsilon_{s}=(\text{1}-D_{T})E_{\text{0}}\varepsilon_{s} \end{array}$$ (14)

Substituting the skeleton-strain expression (Eq. (5)) into Eq. (14), we obtain the relation between $\sigma_{e}$ and $\varepsilon_{\text{1}}$ (still written in terms of $\sigma_{e}$):


$$\begin{array}{c} \sigma_{e}=(\text{1}-D_{T})E_{\text{0}}[\varepsilon_{\text{1}}-\varepsilon_{v}^{c}(\text{1}-exp(-\frac{\sigma_{e}}{\sigma_{c}}))] \end{array}$$ (15)

Then, using the equivalent-stress definition (Eq. (1)) $\sigma_{e}=\sigma_{\text{1}}-\text{2}\nu \sigma_{\text{3}}$in Eq. (15), we get the final $\sigma_{\text{1}}$–$\varepsilon_{\text{1}}$ constitutive relation for Stage I (segment A):


$$\begin{array}{c} \sigma_{\text{1}}-\text{2}\nu \sigma_{\text{3}}=(\text{1}-D_{T})E_{\text{0}}[\varepsilon_{\text{1}}-\varepsilon_{v}^{c}(\text{1}-exp(-\frac{\sigma_{\text{1}}-\text{2}\nu \sigma_{\text{3}}}{\sigma_{c}}))] \end{array}$$ (16)

Equation (16) is an implicit equation (because $\sigma_{\text{1}}$ appears inside the exponential term). In numerical solving, for a given $\varepsilon_{\text{1}}$, $\sigma_{\text{1}}$ can be solved iteratively.

When $\sigma_{e}=\text{0}$ (i.e., $\sigma_{\text{1}}=\text{2}\nu \sigma_{\text{3}}$), Eq. (16) gives $\varepsilon_{\text{1}}=\text{0}$. In addition, the end point of Stage I, $\left(\varepsilon_{\text{1}}^{I},\;\sigma^{I}\right)$, satisfies Eq. (16) only in Case A; the Case B endpoint follows Eq. (31).

Case B: when $\sigma_{e}>\sigma_{e,cc}$, the cracks are basically closed, and the rock-grain skeleton starts to develop stress damage. The stress damage in Stage I is counted from the closure-threshold skeleton strain $\varepsilon_{s,cc}$:


$$\begin{array}{c} D_{I}=\text{0},\text{当}\varepsilon_{s}\leq \varepsilon_{s,cc} \end{array}$$ (17)

$$\begin{array}{c} D_{I}(\varepsilon_{s})=\text{1}-exp(-[\frac{\varepsilon_{s}-\varepsilon_{s},cc}{\varepsilon_{\text{0}}^{I}}{]}^{m_{I}}),\text{当}\varepsilon_{s}>\varepsilon_{s,cc} \end{array}$$ (18)

Here, $\varepsilon_{\text{0}}^{I}$ is the stress-damage scale parameter in Stage I, and $m_{I}$ is the shape parameter. From Eq. (18), we see that $D_{I}=\text{0}$ at $\varepsilon_{s}=\varepsilon_{s,cc}$.

The total accumulated damage from thermal damage and stress damage is taken as: The product is a separability assumption: mechanical damage reduces the fraction remaining after thermal degradation. It permits a compact scalar description but omits explicit interactions, such as thermal defects changing subsequent cyclic damage kinetics [27,39]. Thermal stiffness reduction is counted once through the product with the room-temperature reference modulus; applying the same thermal reduction again to an already thermally reduced modulus would double count it. The combined load-carrying fraction, rather than either component alone, is inherited by later stages. Equivalently, total damage is the sum of the component damages minus their product, which avoids counting their overlapping loss twice. If each component lies between zero and one, the product form preserves this range for total damage. It remains a phenomenological coupling assumption: no additional parameter explicitly describes thermal microcracks accelerating subsequent cyclic damage. The present data are insufficient to identify additional interaction parameters independently.


$$\begin{array}{c} \text{1}-D=(\text{1}-D_{T})(\text{1}-D_{I}) \end{array}$$ (19)

From Eq. (18), we have (for $\varepsilon_{s}>\varepsilon_{s,cc}$):


$$\begin{array}{c} \text{1}-D_{I}=exp(-[\frac{\varepsilon_{s}-\varepsilon_{e}}{\varepsilon_{\text{e}}^{I}}{]}^{m_{I}})(\varepsilon_{s}>\varepsilon_{s,cc}) \end{array}$$ (20)

Substituting Eq. (20) into Eq. (19), we obtain (for $\varepsilon_{s}>\varepsilon_{s,cc}$):


$$\begin{array}{c} \text{1}-D=(\text{1}-D_{T})exp(-[\frac{\varepsilon_{s}-\varepsilon_{s},cc}{\varepsilon_{\text{0}}^{I}}{]}^{m_{I}})(\varepsilon_{s}>\varepsilon_{s,cc}) \end{array}$$ (21)

The skeleton bearing relation is still written in an equivalent-stiffness form:


$$\begin{array}{c} \sigma_{e}=(\text{1}-D)E_{\text{0}}\varepsilon_{s} \end{array}$$ (22)

Substituting Eq. (21) into Eq. (22), we get the explicit skeleton relation for Stage I, Case B:


$$\begin{array}{c} \sigma_{e}=(\text{1}-D_{T})E_{\text{0}}\cdot \varepsilon_{s}\cdot exp(-(\frac{\varepsilon_{s}-\varepsilon_{s,cc}}{\varepsilon_{\text{0}}^{I}}{)}^{m_{I}})(\varepsilon_{s}>\varepsilon_{s,cc}) \end{array}$$ (23)

To obtain a final relation containing only $\sigma_{\text{1}}$ and $\varepsilon_{\text{1}}$, we write the skeleton strain (from Eq. (5), and using Eq. (1) to replace $\sigma_{e}$) as:


$$\begin{array}{c} \varepsilon_{s}=\varepsilon_{\text{1}}-\varepsilon_{v}^{c}[\text{1}-exp(-\frac{\sigma_{\text{1}}-\text{2}\nu \sigma_{\text{3}}}{\sigma_{c}})] \end{array}$$ (24)

Then substituting Eq. (24) into Eq. (23), and using $\sigma_{e}=\sigma_{\text{1}}-\text{2}\nu \sigma_{\text{3}}$, we obtain the final implicit $\sigma_{\text{1}}$–$\varepsilon_{\text{1}}$ constitutive relation for Stage I (segment B):


$$\begin{array}{c} \sigma_{\text{1}}-\text{2}\nu \sigma_{\text{3}}=(\text{1}-D_{T})E_{\text{0}}\cdot \varepsilon_{s}\cdot exp(-[\frac{\varepsilon_{s}-\varepsilon_{s,cc}}{\varepsilon_{\text{0}}^{I}}{]}^{m_{I}}) \end{array}$$ (25)

where $\varepsilon_{s,cc}$ is defined by Eq. (11).

When $\varepsilon_{s}\to \varepsilon_{s,cc}$, the exponential term in Eq. (23) tends to exp(0)=1, so$\sigma_{e}\to (\text{1}-D_{T})E_{\text{0}}\varepsilon_{s}=E_{T}\varepsilon_{s},$

which is consistent with Eq. (14) in Case A. Therefore, segments A and B connect continuously at the threshold point.

The end point of Stage I (i.e., the starting point of cyclic loading) is denoted as:


$$\begin{array}{c} \left(\varepsilon_{\text{1}},\sigma_{\text{1}})=(\varepsilon^{I},\sigma^{I}\right) \end{array}$$ (26)

The corresponding equivalent stress is:


$$\begin{array}{c} \sigma_{e}^{I}=\sigma^{I}-\text{2}\nu \sigma_{\text{3}} \end{array}$$ (27)

The compaction strain at this point can be obtained from Eq. (4):


$$\begin{array}{c} \varepsilon_{v}^{I}=\varepsilon_{v}^{c}[\text{1}-exp(-\frac{\sigma_{e}^{I}}{\sigma_{c}})] \end{array}$$ (28)

From Eq. (3), the skeleton strain at the end point is:


$$\begin{array}{c} \varepsilon_{s}^{I}=\varepsilon^{I}-\varepsilon_{v}^{I} \end{array}$$ (29)

If $\sigma_{e}^{I}\leq \sigma_{e,cc}$, the end point lies in Case A, and it should satisfy the linear skeleton relation (Eq. (14)):


$$\begin{array}{c} \sigma_{e}^{I}=E_{T}\varepsilon_{s}^{I} \end{array}$$ (30)

If $\sigma_{e}^{I}>\sigma_{e,cc}$, the end point lies in Case B. Substituting $\varepsilon_{s}=\varepsilon_{s}^{I}$ and $\sigma_{e}=\sigma_{e}^{I}$ into Eq. (23), we get:


$$\begin{array}{c} \sigma_{e}^{I}=(\text{1}-D_{T})E_{\text{0}}\cdot \varepsilon_{s}^{I}\cdot exp(-[\frac{\varepsilon_{s}^{I}-\varepsilon_{s,cc}}{\varepsilon_{\text{0}}^{I}}{]}^{m_{I}}) \end{array}$$ (31)

To solve $\varepsilon_{\text{0}}^{I}$ explicitly, we first define a dimensionless ratio:


$$\begin{array}{c} Q=\frac{\sigma_{e}^{I}}{(\text{1}-D_{T})E_{\text{0}}\varepsilon_{s}^{I}} \end{array}$$ (32)

Substituting Eq. (32) into Eq. (31), we obtain:


$$\begin{array}{c} exp(-[\frac{\varepsilon_{s}^{I}-\varepsilon_{s,cc}}{\varepsilon_{\text{0}}^{I}}{]}^{m_{I}})=Q \end{array}$$ (33)

When 0<Q<1, the explicit expression of $\varepsilon_{\text{0}}^{I}$ is:


$$\begin{array}{c} \varepsilon_{\text{0}}^{I}=\frac{\left(\varepsilon_{s}^{I}-\varepsilon_{s,cc}\right)}{{\left[-ln(Q)\right]}^{\left(\frac{\text{1}}{m_{I}}\right)}} \end{array}$$ (34)

The final constitutive model of Stage I is:

Case A ($\sigma_{e}\leq \sigma_{e,cc}$):


$$\begin{array}{c} \sigma_{\text{1}}-\text{2}\nu \sigma_{\text{3}}=(\text{1}-D_{T})E_{\text{0}}[\varepsilon_{\text{1}}-\varepsilon_{v}^{c}(\text{1}-exp(-\frac{\sigma_{\text{1}}-\text{2}\nu \sigma_{\text{3}}}{\sigma_{c}}))] \end{array}$$ (35)

Case B ($\sigma_{e}>\sigma_{e,cc}$):


$$\begin{array}{c} \sigma_{\text{1}}-\text{2}\nu \sigma_{\text{3}}=(\text{1}-D_{T})E_{\text{0}}\cdot \varepsilon_{s}\cdot exp(-[\frac{\varepsilon_{s}-\varepsilon_{s,cc}}{\varepsilon_{\text{0}}^{I}}{]}^{m_{I}}) \end{array}$$ (36)

where:


$$\begin{array}{c} \varepsilon_{s}=\varepsilon_{\text{1}}-\varepsilon_{v}^{c}[\text{1}-exp(-\frac{\sigma_{\text{1}}-\text{2}\nu \sigma_{\text{3}}}{\sigma_{c}})] \end{array}$$ (37)

$$\begin{array}{c} \varepsilon_{s,cc}=\varepsilon_{cc}-\varepsilon_{v}^{c}[\text{1}-exp(-\frac{\sigma_{cc}-\text{2}\nu \sigma_{\text{3}}}{\sigma_{c}})] \end{array}$$ (38)

### 4.2. Stage II: Graded cyclic stress part

$D_{T}$ thermal damage, which shows how much stiffness is reduced by high temperature. It is defined as:


$$\begin{array}{c} D_{T}=\text{1}-\frac{E_{T}}{E_{\text{0}}} \end{array}$$ (39)

To simplify the formulas, a coefficient $A_{k}$ that depends only on the loading history is introduced. It is given by


$$\begin{array}{c} A_{k}=(\text{1}-D_{k-\text{1}})E_{\text{0}} \end{array}$$ (40)

Here, $E_{k}$ represents the equivalent modulus at the start of cycle k, and $\text{1}-D_{k-\text{1}}$ is the total load-carrying ratio at the start of cycle k. It reflects how the damage accumulated in the previous cycle affects the current loading. The history coefficient and the equivalent modulus represent the same inherited stiffness in Eqs. (40) and (49). The residual-strain reference is the measured axial strain at the preceding unloading endpoint, not a residual strain independently predicted by the model. At the loading peak, Eqs. (57),(58) define the updated total damage used in the unloading expression. These definitions identify the state quantities that must be carried between cycles; the stress-level change is not a new thermal treatment. At the end of cycle k, the measured state is εr,k = εexp,unload,end,k. This state and the updated damage/load-carrying state initialize cycle k + 1. The final states at Levels I and II likewise initialize the first cycles at Levels II and III, respectively; neither residual strain nor damage is reset at a level change. This is a semi-empirical update driven by experimentally determined states. Energy drives mechanical damage evolution, whereas strain equivalence maps the inherited damage to stiffness: the mechanical remaining fraction is *η* = 1 − *D*_σ_, the total remaining fraction is 1 −* D* = (1 − *D*_T_)η, and the equivalent modulus is *E* = (1 − *D*)*E*_0_. Accumulation is represented through state inheritance, not arithmetic addition of cycle damages.

In this constitutive model, energy density is the key factor that drives the growth of stress damage. First, we define the energy density in the loading part of cycle k, from the residual strain $\varepsilon_{r,k-\text{1}}$to the current strain $\varepsilon_{\text{1}}$, as


$$\begin{array}{c} W_{k}=\sum_{k=\text{1}}^{n}u_{k}(\varepsilon_{\text{1}}) \end{array}$$ (41)

where $u_{k}(\varepsilon_{\text{1}})$ is the energy contribution used to drive the phenomenological damage law during one cyclic loading process; this model quantity should not be interpreted as proof that all measured hysteresis dissipation is consumed by damage.

Next, the evolution of stress damage $D_{\sigma }$ is related to the energy density $W_{k}$. A phenomenological energy–damage relation is assumed; it is not derived here from a specified free-energy potential and dissipation inequality. The proposed relation is


$$\begin{array}{c} \frac{d(\text{1}-D_{\sigma })}{dW_{k}}=-\frac{\left(\text{1}-D_{\sigma }\right)}{W_{f}} \end{array}$$ (42)

where $W_{f}$ is the characteristic energy density, which gives the energy scale of the damage process. It has the same units as the driving energy density. In the exponential law, a larger positive scale gives slower mechanical degradation for the same driving energy. It is not a finite failure energy at which damage reaches one, since the exponential law approaches that limit asymptotically. Physically, it is associated with energy absorbed up to peak failure under the same temperature and confinement conditions and may depend on temperature; no universal value across treatments is implied. The Stage III energy scale reported in Table 4 is a separate parameter and is not substituted for Stage II $W_{f}$; an independently confirmed Stage II value and its mapping to the numerical implementation remain unavailable.

**Table 4:** Stage III characteristic energy density by temperature.

Temperature (°C)	$W_{f}^{\left(III\right)}$ (MJ/m³)
25 °C	1.8538
300 °C	4.7154
600 °C	2.9986
900 °C	3.2624

$W_{fIII}$ denotes the Stage III parameter in Eqs. (89)–(97); these values do not supply or replace Stage II $W_{f}$.

From this, the relation between stress damage $D_{\sigma }$ and energy density $W_{k}$ is


$$\begin{array}{c} D_{\sigma }=\text{1}-\text{exp}(-\frac{W_{k}}{W_{f}}) \end{array}$$ (43)

where $D_{\sigma }$ is stress damage, and $\text{1}-D_{\sigma }$ is the mechanical load-carrying ratio of the rock, showing how its bearing capacity changes during loading. The exponential form in Eq. (43) corresponds mathematically to a constant fractional loss of the mechanical load-carrying ratio per increment of the driving energy. For a positive energy scale and nonnegative driving energy, it is positive and decreases monotonically. A larger characteristic energy scale produces slower decay. This explains the choice of functional form without establishing it as a unique microscopic damage law or equating the energy scale to the measured loss in a single cycle.

In the cyclic loading stage, the stress–strain relation for the loading branch of cycle k is established by considering the coupling between energy density and stress damage, from which an explicit constitutive model is obtained.

The “frame” form of the loading formula is the same as the original one. But since damage evolves with energy and cycling, we define


$$\begin{array}{c} \sigma_{\text{1}}-\text{2}\nu \sigma_{\text{3}}=(\text{1}-D)E_{\text{0}}(\varepsilon_{\text{1}}-\varepsilon_{r,k-\text{1}}) \end{array}$$ (44)

where $(\text{1}-D)E_{\text{0}}$ is the current elastic modulus, and $\varepsilon_{\text{1}}-\varepsilon_{r,k-\text{1}}$ is the effective loading strain relative to the residual strain.

Since the total damage includes only thermal damage $D_{T}$ and stress damage $D_{\sigma }$, and they are combined by a product-type coupling, the starting damage of cycle k is


$$\begin{array}{c} \text{1}-D_{k-\text{1}}=(\text{1}-D_{T})(\text{1}-D_{\sigma ,k-\text{1}}) \end{array}$$ (45)

Using this in the loading relation gives


$$\begin{array}{c} \sigma_{\text{1}}-\text{2}\nu \sigma_{\text{3}}=(\text{1}-D_{T})(\text{1}-D_{\sigma })E_{\text{0}}(\varepsilon_{\text{1}}-\varepsilon_{r,k-\text{1}}) \end{array}$$ (46)

Then we solve Eqs. (42) and (41) together. For easier calculation, define


$$\begin{array}{c} x=\varepsilon_{\text{1}}-\varepsilon_{r,k-\text{1}} \end{array}$$ (47)

So, the closed-form solution of energy density during loading is


$$\begin{array}{c} W_{k}=W_{f}\text{ln}\left(\text{1}+\frac{E_{k}}{\text{2}W_{f}}x^{\text{2}}\right) \end{array}$$ (48)

With this, the final explicit nonlinear constitutive relation for the loading branch is


$$\begin{array}{c} \sigma_{\text{1}}(\varepsilon_{\text{1}})=\text{2}\nu \sigma_{\text{3}}+\frac{E_{k}x}{\text{1}+\frac{A_{k}}{\text{2}W_{f}}x^{\text{2}}} \end{array}$$ (49)

where $x=\varepsilon_{\text{1}}-\varepsilon_{r,k-\text{1}}$, and $E_{k}=(\text{1}-D_{k-\text{1}})E_{\text{0}}$. The residual-strain reference is the measured minimum-stress unloading endpoint. It is not defined by zero equivalent stress. The confinement offset in Eq. (2) is a constitutive idealization and does not redefine the experimental endpoint. With the present expressions, zero incremental strain gives the confinement-offset stress; therefore, state inheritance alone does not establish exact stress matching to the measured lower endpoint.

From Eqs. (44) and (47), the damage expression can be written as


$$\begin{array}{c} \text{1}-D(\varepsilon_{\text{1}})=\frac{\text{1}-D_{k-\text{1}}}{\text{1}+\frac{E_{k}}{\text{2}W_{f}}(\varepsilon_{\text{1}}-\varepsilon_{r,k-\text{1}}{)}^{\text{2}}} \end{array}$$ (50)

For the unloading branch of cycle k, the stress–strain relation is first defined by the tangent modulus:


$$\begin{array}{c} \frac{d(\sigma_{\text{1}}-\text{2}\nu \sigma_{\text{3}})}{d\varepsilon_{\text{1}}}=(\text{1}-D_{k})E_{\text{0}}\left(\text{1}+\frac{\sigma_{\text{1}}-\text{2}\nu \sigma_{\text{3}}}{\sigma_{\text{1},p}-\text{2}\nu \sigma_{\text{3}}}\right) \end{array}$$ (51)

Where $\sigma_{\text{1},p}$: the plateau peak axial total stress in the current cycle, $D_{k}$: the total damage in cycle k.

Integrating Eq. (51) with the residual-strain reference, we obtain the final explicit unloading expression (only in terms of $\sigma_{\text{1}}$ and $\varepsilon_{\text{1}}$):


$$\begin{array}{c} \sigma_{\text{1}}(\varepsilon_{\text{1}})=\text{2}\nu \sigma_{\text{3}}+(\sigma_{\text{1},p}-\text{2}\nu \sigma_{\text{3}})\left(\text{exp}\left[\frac{(\text{1}-D_{k})E_{\text{0}}}{\sigma_{\text{1},p}-\text{2}\nu \sigma_{\text{3}}}(\varepsilon_{\text{1}}-\varepsilon_{r,k})\right]-\text{1}\right) \end{array}$$ (52)

First define the strain increment at the peak point:


$$\begin{array}{c} x_{p}=\varepsilon_{\text{1},p}-\varepsilon_{r,k-\text{1}} \end{array}$$ (53)

The peak point satisfies the loading formula (Eq. (49)) when $\sigma_{\text{1}}=\sigma_{\text{1},p}$:


$$\begin{array}{c} \sigma_{\text{1},p}-\text{2}\nu \sigma_{\text{3}}=\frac{Ex_{p}}{\text{1}+\frac{A_{k}}{\text{2}W_{f}}x_{p}^{\text{2}}} \end{array}$$ (54)

So we can obtain


$$\begin{array}{c} x_{p}=\frac{W_{f}}{\sigma_{\text{1},p}-\text{2}\nu \sigma_{\text{3}}}\left[\text{1}-\sqrt{\text{1}-\frac{\text{2}(\sigma_{\text{1},p}-\text{2}\nu \sigma_{\text{3}}{)}^{\text{2}}}{W_{f}E_{k}}}\right] \end{array}$$ (55)

From Eq. (47), at the peak point we have


$$\begin{array}{c} u_{k,p}=W_{f}\text{ln}\left(\text{1}+\frac{E_{k}}{\text{2}W_{f}}x_{p}^{\text{2}}\right) \end{array}$$ (56)

The decay of the load-carrying ratio driven by energy gives


$$\begin{array}{c} \text{1}-D_{k}=\frac{\text{1}-D_{k-\text{1}}}{\text{1}+\frac{E_{k}}{\text{2}W_{f}}x_{p}^{\text{2}}} \end{array}$$ (57)

Therefore,


$$\begin{array}{c} D_{k}=\text{1}-\frac{\text{1}-D_{k-\text{1}}}{\text{1}+\frac{E_{k}}{\text{2}W_{f}}x_{p}^{\text{2}}} \end{array}$$ (58)

So, the final constitutive model for cycle k is:

Loading branch


$$\begin{array}{c} \sigma_{\text{1}}(\varepsilon_{\text{1}})=\text{2}\nu \sigma_{\text{3}}+\frac{\left(\text{1}-D_{k-\text{1}})E_{\text{0}}(\varepsilon_{\text{1}}-\varepsilon_{r,k-\text{1}}\right)}{\text{1}+\frac{(\text{1}-D_{k-\text{1}})E_{\text{0}}}{\text{2}W_{f}}(\varepsilon_{\text{1}}-\varepsilon_{r,k-\text{1}}{)}^{\text{2}}} \end{array}$$ (59)

Unloading branch


$$\begin{array}{c} \sigma_{\text{1}}(\varepsilon_{\text{1}})=\text{2}\nu \sigma_{\text{3}}+(\sigma_{\text{1},p}-\text{2}\nu \sigma_{\text{3}})(\text{exp}\left[\frac{(\text{1}-D_{k})E_{\text{0}}}{\sigma_{\text{1},p}-\text{2}\nu \sigma_{\text{3}}}(\varepsilon_{\text{1}}-\varepsilon_{r,k})\right]-\text{1}) \end{array}$$ (60)

### 4.3. Stage III: Monotonic loading

First, define the incremental strain $\bar{\varepsilon }$relative to the residual strain as


$$\begin{array}{c} \overset{―}{\varepsilon }=\varepsilon_{\text{1}}-\varepsilon_{r} \end{array}$$ (61)

where $\varepsilon_{\text{1}}$ is the total axial strain, $\varepsilon_{r}$ is the residual strain after cyclic loading, and $\bar{\varepsilon }$ is the incremental strain used in Stage III. The initial total damage and residual-strain reference in Stage III are inherited from the final cyclic state, rather than resetting the specimen to an undamaged state. The residual reference is the measured strain at the final unloading minimum; the corresponding measured axial stress and total strain identify the experimental transition. Both Stage III alternatives use zero incremental strain at this reference and inherit the final total load-carrying ratio. Their confinement-offset stress intercept retains the endpoint-matching limitation noted in Section 4.2 it is not a redefinition of the measured residual state.

The statistical damage in this stage is defined as


$$\begin{array}{c} D_{m}(\bar{\varepsilon })=\text{1}-\text{exp}(-{\left(\frac{\bar{\varepsilon }}{\varepsilon_{\text{0}}}\right)}^{m}) \end{array}$$ (62)

where $D_{m}(\bar{\varepsilon })$ is the new statistical damage added in Stage III, and $\varepsilon_{\text{0}}$ and m are the parameters of the Weibull distribution.

The total damage is combined by the post-cycling initial damage $D_{c}$ and the new damage $D_{m}(\bar{\varepsilon })$ in the remaining intact part:


$$\begin{array}{c} D\left(\bar{\varepsilon }\right)=D_{c}+\left(\text{1}-D_{c}\right)D_{m}\left(\bar{\varepsilon }\right) \end{array}$$ (63)

Since 1−D is the current load-carrying ratio, we have


$$\begin{array}{c} \text{1}-D(\bar{\varepsilon })=(\text{1}-D_{c})\text{exp}(-{\left(\frac{\bar{\varepsilon }}{\varepsilon_{\text{0}}}\right)}^{m}) \end{array}$$ (64)

Next, define the load-bearing component (equivalent stress) as


$$\begin{array}{c} \sigma_{e}=\sigma_{\text{1}}-\text{2}\nu \sigma_{\text{3}} \end{array}$$ (65)

where $\sigma_{\text{1}}$ is the total axial stress, $\sigma_{\text{3}}$ is the confining pressure, and νis Poisson’s ratio.

Based on the equivalent-strain assumption, the damage elastic constitutive relation is


$$\begin{array}{c} \sigma_{e}=(\text{1}-D)E_{\text{0}}\bar{\varepsilon } \end{array}$$ (66)

where $E_{\text{0}}$ is the reference elastic modulus of the pre-dried 25 °C reference specimen, and D is the total damage.

Substituting Eq. (64) into Eq. (66), we obtain the load-bearing component in Stage III:


$$\begin{array}{c} \sigma_{e}(\varepsilon_{\text{1}})=(\text{1}-D_{c})E_{\text{0}}(\varepsilon_{\text{1}}-\varepsilon_{r})\text{exp}(-{\left(\frac{\varepsilon_{\text{1}}-\varepsilon_{r}}{\varepsilon_{\text{0}}}\right)}^{m}) \end{array}$$ (67)

Then, using Eq. (65) to recover the total axial stress $\sigma_{\text{1}}$, we get


$$\begin{array}{c} \sigma_{\text{1}}(\varepsilon_{\text{1}})=\text{2}\nu \sigma_{\text{3}}+(\text{1}-D_{c})E_{\text{0}}(\varepsilon_{\text{1}}-\varepsilon_{r})\text{exp}(-{\left(\frac{\varepsilon_{\text{1}}-\varepsilon_{r}}{\varepsilon_{\text{0}}}\right)}^{m}) \end{array}$$ (68)

This is the constitutive relation for Stage III.

The peak point on the stress–strain curve is assumed to satisfy


$$\begin{array}{c} \sigma_{\text{1}}(\varepsilon_{\text{1}}=\varepsilon_{p})=\sigma_{p} \end{array}$$ (69)

and


$$\begin{array}{c} {\left(\frac{d\sigma_{\text{1}}}{d\varepsilon_{\text{1}}}\right)}_{\varepsilon_{\text{1}}=\varepsilon_{p}}=\text{0} \end{array}$$ (70)

where $\varepsilon_{p}$is the experimental peak strain and $\sigma_{p}$ is the experimental peak stress.

Define the peak incremental strain as


$$\begin{array}{c} \varepsilon_{\bar{p}}=\varepsilon_{p}-\varepsilon_{r} \end{array}$$ (71)

To make the calculation easier, define


$$\begin{array}{c} z={\left(\frac{\varepsilon_{\text{1}}-\varepsilon_{r}}{\varepsilon_{\text{0}}}\right)}^{m} \end{array}$$ (72)

Differentiating Eq. (68) gives


$$\begin{array}{c} \frac{d\sigma_{\text{1}}}{d\varepsilon_{\text{1}}}=(\text{1}-D_{c})E_{\text{0}}\text{exp}[-z][\text{1}-mz] \end{array}$$ (73)

At the peak point $\varepsilon_{\text{1}}=\varepsilon_{p}$, applying Eq. (70) yields


$$\begin{array}{c} \text{1}-mz_{p}=\text{0}\Rightarrow z_{p}=\frac{\text{1}}{m} \end{array}$$ (74)

From Eq. (72), we obtain


$$\begin{array}{c} {\left(\frac{\varepsilon_{\bar{p}}}{\varepsilon_{\text{0}}}\right)}^{m}=\frac{\text{1}}{m} \end{array}$$ (75)

Solving Eq. (75) for $\varepsilon_{\text{0}}$ gives


$$\begin{array}{c} \varepsilon_{\text{0}}=\varepsilon {\bar{\;}}_{p}m^{\frac{\text{1}}{m}}=(\varepsilon_{p}-\varepsilon_{r})m^{\frac{\text{1}}{m}} \end{array}$$ (76)

Substituting $\varepsilon_{p}$ into Eq. (68), and using the peak constraint (Eq. (74)), we obtain


$$\begin{array}{c} \sigma_{p}-\text{2}\nu \sigma_{\text{3}}=(\text{1}-D_{c})E_{\text{0}}(\varepsilon_{p}-\varepsilon_{r})\text{exp}(-\frac{\text{1}}{m}) \end{array}$$ (77)

For convenience, define a dimensionless ratio


$$\begin{array}{c} R=\frac{\sigma_{p}-\text{2}\nu \sigma_{\text{3}}}{\left(\text{1}-D_{c})E_{\text{0}}(\varepsilon_{p}-\varepsilon_{r}\right)} \end{array}$$ (78)

From Eq. (77), we get


$$\begin{array}{c} \text{exp}(-\frac{\text{1}}{m})=R \end{array}$$ (79)

Thus,


$$\begin{array}{c} m=-\frac{\text{1}}{\text{ln}(R)} \end{array}$$ (80)

From Eq. (76), we also have


$$\begin{array}{c} \varepsilon_{\text{0}}=\frac{\left(\varepsilon_{p}-\varepsilon_{r}\right)}{m^{\frac{\text{1}}{m}}} \end{array}$$ (81)

Therefore, the final constitutive formula is


$$\begin{array}{c} \sigma_{\text{1}}(\varepsilon_{\text{1}})=\text{2}\nu \sigma_{\text{3}}+(\text{1}-D_{c})E_{\text{0}}(\varepsilon_{\text{1}}-\varepsilon_{r})\text{exp}[-{\left(\frac{\varepsilon_{\text{1}}-\varepsilon_{r}}{\varepsilon_{\text{0}}}\right)}^{m}] \end{array}$$ (82)

where m and $\varepsilon_{\text{0}}$ are given by Eqs. (79)–(81). The corresponding theoretical and experimental curves are compared in Fig 9.

**Fig 9:**
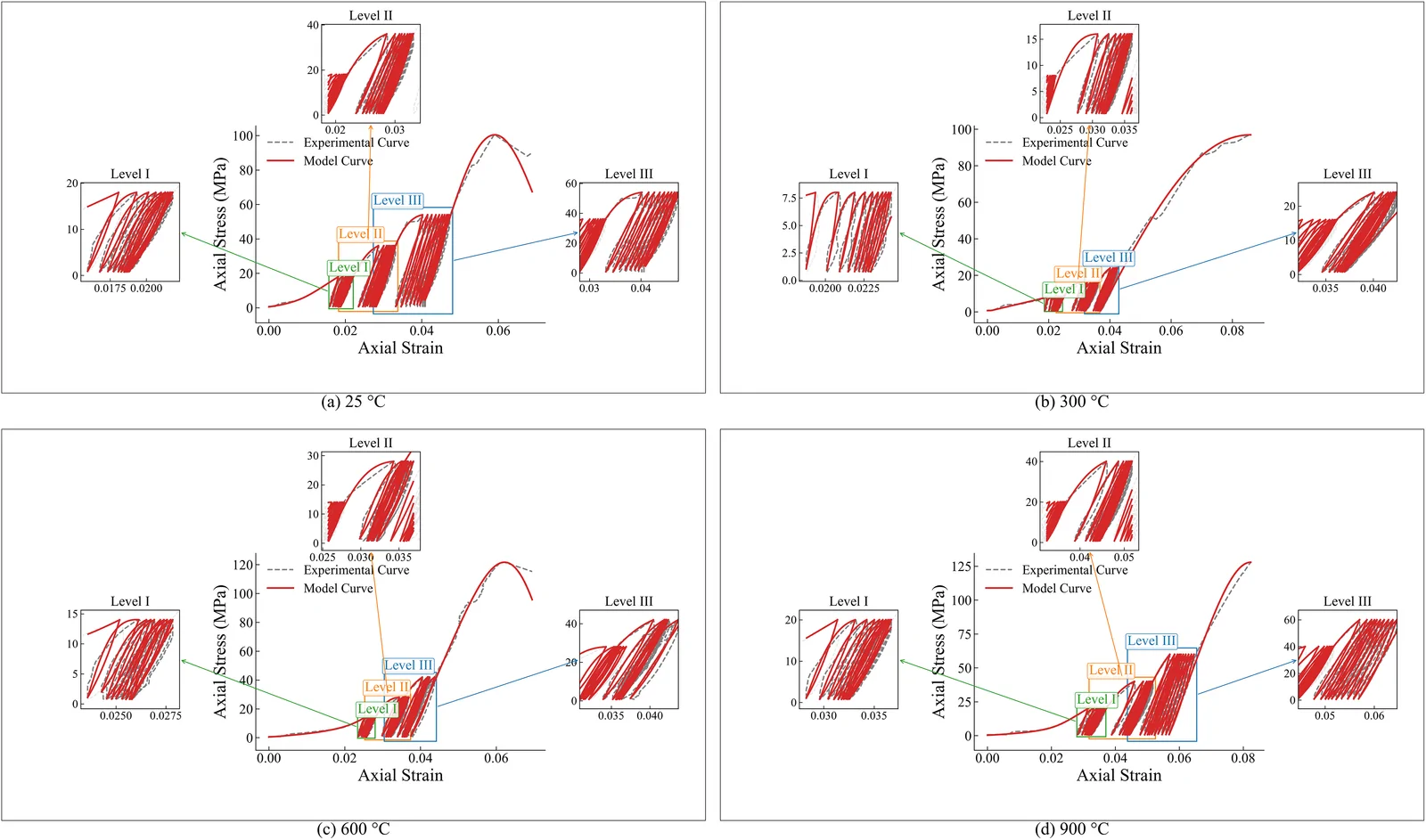
Comparison between theoretical curves and experimental curves at different temperatures in Stage III under the Weibull distribution.

After substituting $\varepsilon_{\text{0}}$ from Eq. (76) into Eq. (82), we obtain the alternative final form:


$$\begin{array}{c} \sigma_{\text{1}}(\varepsilon_{\text{1}})=\text{2}\nu \sigma_{\text{3}}+(\text{1}-D_{c})E_{\text{0}}(\varepsilon_{\text{1}}-\varepsilon_{r})\text{exp}(-\frac{\text{1}}{m}{\left[\frac{\varepsilon_{\text{1}}-\varepsilon_{r}}{\varepsilon_{p}-\varepsilon_{r}}\right]}^{m}) \end{array}$$ (83)

where m is determined by the peak-point constraints (Eqs. (69)–(70)):


$$\begin{array}{c} m=-\frac{\text{1}}{\text{ln}\left(\frac{\sigma_{p}-\text{2}\nu \sigma_{\text{3}}}{\left(\text{1}-D_{c})E_{\text{0}}(\varepsilon_{p}-\varepsilon_{r}\right)}\right)} \end{array}$$ (84)

### 4.4. Stage III: Monotonic damage constitutive derivation driven by energy density

Relative to the residual strain after cyclic loading, the Stage III incremental strain is defined as


$$\begin{array}{c} \bar{\varepsilon }=\varepsilon_{\text{1}}-\varepsilon_{r} \end{array}$$ (85)

To keep the same equivalent-stress framework as before, the load-bearing component is defined as


$$\begin{array}{c} \sigma_{e}=\sigma_{\text{1}}-\text{2}\nu \sigma_{\text{3}} \end{array}$$ (86)

In the monotonic loading segment, the work per unit volume counted from the start of Stage III ($\bar{\varepsilon }=\text{0}$) is defined as an internal energy-density variable: This is the loading work associated with equivalent stress, measured from the Stage III strain origin. It is not automatically identical to the hysteresis loss reported for a loading–unloading cycle in Section 3.2. The Stage III energy origin can be local while the damage state retains the preceding cyclic history through Eq. (90).


$$\begin{array}{c} u(\bar{\varepsilon })=\int_{\text{0}}^{\bar{\varepsilon }}\sigma_{e}(\xi )\;d\xi \end{array}$$ (87)

Therefore,


$$\begin{array}{c} \frac{dW}{d\bar{\varepsilon }}=\sigma_{e} \end{array}$$ (88)

Let the load-carrying ratio Y=1−D decay exponentially under the driving of energy density:


$$\begin{array}{c} \frac{dY}{dW}=-\frac{Y}{W_{f}^{\left(III\right)}} \end{array}$$ (89)

Where $W=\int_{\text{0}}^{\bar{\varepsilon }}\sigma_{e}\;d\bar{\varepsilon },$ and $W_{f}^{\left(\text{III}\right)}$ is the characteristic energy-density parameter in Stage III. The start point of Stage III inherits the initial value from the cyclic history:


$$\begin{array}{c} Y(\text{0})=\text{1}-D_{c} \end{array}$$ (90)

Using the damage-elastic closure relation under the strain equivalence assumption:


$$\begin{array}{c} \sigma_{e}=YE_{\text{0}}\bar{\varepsilon } \end{array}$$ (91)

From Eqs. (88), (89), and (91), we obtain


$$\begin{array}{c} \frac{dY}{d\bar{\varepsilon }}=\frac{dY}{dW}\frac{dW}{d\bar{\varepsilon }}=(-\frac{Y}{W_{f}^{\left(III\right)}})\sigma_{e}=-\frac{E_{\text{0}}}{W_{f}^{\left(III\right)}}Y^{\text{2}}\bar{\varepsilon } \end{array}$$ (92)

Separating variables in Eq. (92) and integrating with the initial value (Eq. (90)), we get the closed-form solution of the load-carrying ratio:


$$\begin{array}{c} Y(\bar{\varepsilon })=\frac{\text{1}-D_{c}}{\text{1}+\frac{(\text{1}-D_{c})E_{\text{0}}}{\text{2}W_{f}^{\left(III\right)}}{\bar{\varepsilon }}^{\text{2}}} \end{array}$$ (93)

Substituting Eq. (93) into Eq. (91) gives the explicit equivalent-stress expression:


$$\begin{array}{c} \sigma_{e}(\bar{\varepsilon })=\frac{(\text{1}-D_{c})E_{\text{0}}\bar{\varepsilon }}{\text{1}+\frac{(\text{1}-D_{c})E_{\text{0}}}{\text{2}W_{f}^{\left(III\right)}}{\bar{\varepsilon }}^{\text{2}}} \end{array}$$ (94)

Using Eqs. (85) and (86) to return to the total axial stress–total axial strain form, the final explicit constitutive model of Stage III is


$$\begin{array}{c} \sigma_{\text{1}}(\varepsilon_{\text{1}})=\text{2}\nu \sigma_{\text{3}}+\frac{\left(\text{1}-D_{c})E_{\text{0}}(\varepsilon_{\text{1}}-\varepsilon_{r}\right)}{\text{1}+\frac{(\text{1}-D_{c})E_{\text{0}}}{\text{2}W_{f}^{\left(III\right)}}(\varepsilon_{\text{1}}-\varepsilon_{r}{)}^{\text{2}}} \end{array}$$ (95)

From the peak constraint ${\frac{d\sigma_{\text{1}}}{d\varepsilon_{\text{1}}}|}_{\varepsilon_{\text{1}}=\varepsilon_{p}}=\text{0},$ the peak incremental strain is obtained as


$$\begin{array}{c} \varepsilon_{p}-\varepsilon_{r}=\sqrt{\frac{\text{2}W_{f}^{\left(III\right)}}{(\text{1}-D_{c})E_{\text{0}}}} \end{array}$$ (96)

Then, using $\sigma_{e,p}=\sigma_{p}-\text{2}\nu \sigma_{\text{3}}$, the Stage III energy scale is obtained from Eq. (97). This links the characteristic energy scale to the peak stress and inherited stiffness within this formulation. Treating an energy scale as constant within a branch is a modeling assumption and does not establish that the same value applies across temperatures or damage histories. The theoretical and experimental Stage III curves are compared in Fig 10. Table 4 reports the temperature-specific Stage III characteristic energy densities associated with this formulation.


$$\begin{array}{c} W_{f}^{\left(III\right)}=\frac{\text{2}(\sigma_{p}-\text{2}\nu \sigma_{\text{3}}{)}^{\text{2}}}{(\text{1}-D_{c})E_{\text{0}}} \end{array}$$ (97)

**Fig 10:**
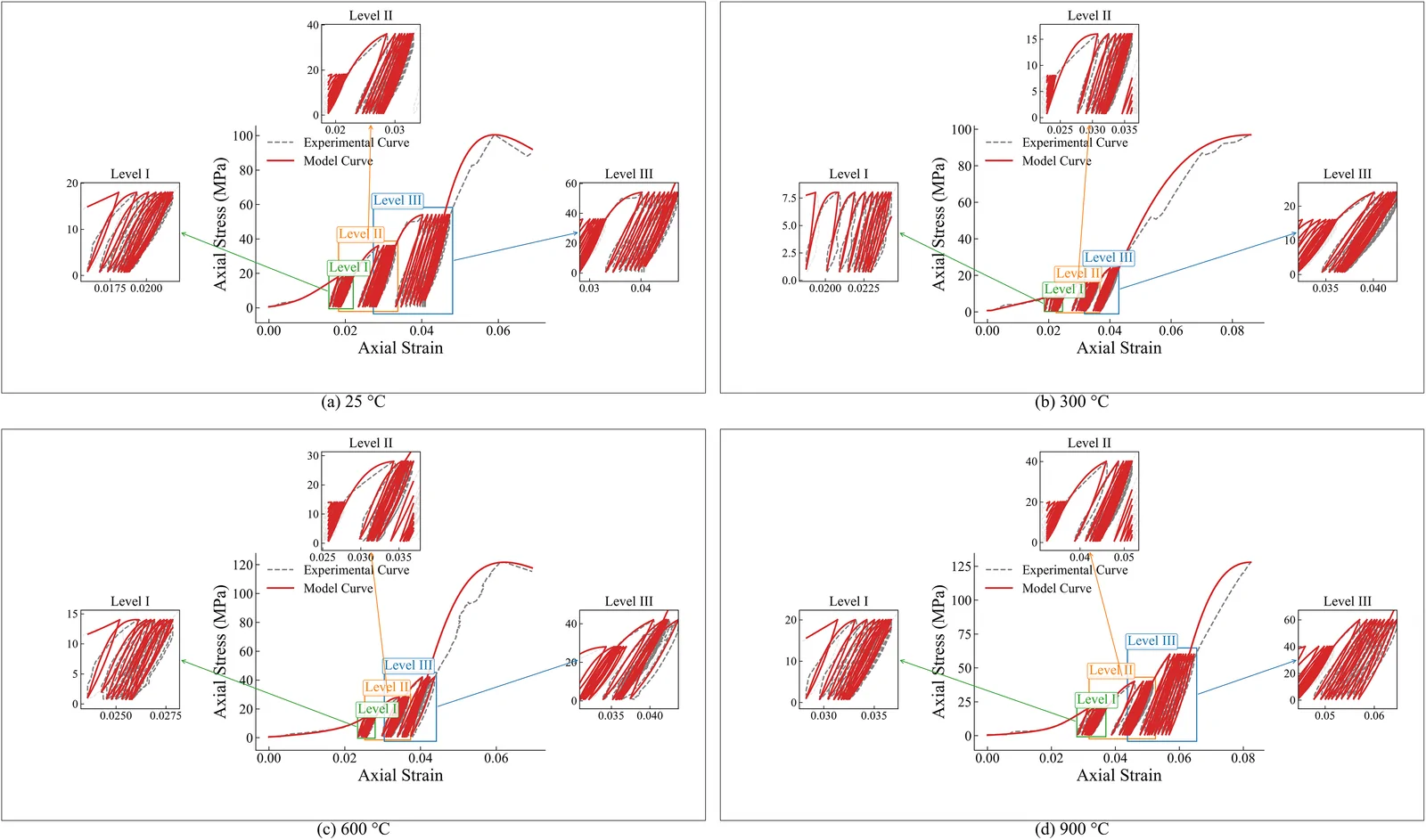
Comparison between theoretical curves and experimental curves at different temperatures in Stage III under the energy-driven model.

## 5. Discussion

### 5.1. Existing models and the comparison framework

The models listed in Table 1 cover several technical routes, ranging from “mainly empirical cumulative damage evolution”, to “recursive/incremental reconstruction of cyclic curves”, and then to “damage models that explicitly include structural effects or energy variables”. For each type, the application scenarios, strengths, and limits have been sorted in a matched way. All these models aim to reproduce strength and stiffness degradation, residual strain accumulation, and hysteresis features under multi-level cycles or complex paths. However, they make different trade-offs among “accuracy–usability–number of parameters–computing efficiency”. Empirical models and some three-stage evolution models usually have simpler forms and faster calibration, so they are convenient for quick engineering use, but their transfer to different rock types or different loading paths often depends on re-calibration. Recursive/incremental frameworks can reconstruct hysteresis and stiffness loss more directly and are friendly for numerical implementation, but their “damage state” sometimes acts more like a curve-update rule than a unified internal variable. Models that couple structure or include energy variables usually give stronger mechanism explanations and are more sensitive to path effects, but they need more parameters and more data, and their computation chain is longer.

From the full path considered in this paper (“temperature treatment + multi-level cycles + final crushing stage”), Table 1 also points to a key gap: many studies still focus on macro index comparison or peak fitting. They lack a unified and practical framework to support (i) the step-by-step evolution of energy input/storage/dissipation during multi-level cycles, (ii) the energy jumps caused by stress-level switching, and (iii) the connection to the subsequent monotonic response. This gap is also the main motivation for this paper to stress the consistency of “energy–damage–path” in constitutive expressions.

### 5.2. Energy-Driven recursive framework in this paper

To put path effects and energy information of multi-level cycles into one logic chain, this paper first makes two “separation” treatments in the mechanical expression. First, the effect of confining pressure on axial response is treated as a constant offset in the axial load-bearing part, and an equivalent stress is introduced so that later nonlinear mechanisms can be written in a unified way on this load-bearing part. Second, the total strain is split into skeleton strain and an extra compaction strain caused by pore/crack closure. This avoids mixing compaction nonlinearity with damage evolution in the math form, and makes stage division and parameter identification more workable. In the cyclic stage (Stage II), the model further takes energy density as the key driver of stress-damage evolution. By combining the energy-density definition, an assumed energy–damage relation, and the load-carrying ratio expression, an explicit constitutive relation that can be updated in a recursive way is obtained. In this way, the influence of cyclic history on the current cycle response can enter the update in the form of “history state variables”. The residual-strain input is taken from the measured unloading endpoints, so this update reproduces a response conditioned on experimental states and does not independently predict residual deformation.

The recursive framework provides a phenomenological way to relate loading history to the current constitutive response. The observed energy jumps at stress-level transitions motivate this representation, but they do not independently establish the assumed energy–damage law. Its reproducibility depends on unambiguous energy definitions, state-transfer conventions and parameter identification; the characteristic energy parameter must not be treated as a directly measured hysteresis loss without establishing that correspondence.

### 5.3. Stage III: Comparison of weibull statistical damage and energy-density-driven damage

For the monotonic loading segment after cycling (Stage III), this paper provides two damage paths that can be implemented in parallel. One is the Weibull-based statistical damage form. It builds total damage by combining “post-cycling initial damage + new statistical damage in the remaining intact part”, and then uses the load-carrying ratio and the equivalent-strain assumption to obtain the Stage III constitutive relation. Key parameters are then back-calculated using the peak-point constraints. The other is the energy-density-driven form. It defines the work per unit volume counted from the start of Stage III as an internal energy-density variable, and lets the load-carrying ratio decay under energy driving, which leads to a closed-form solution for the load-carrying ratio and an explicit total stress–total strain expression.

In terms of final results, the Weibull form is often easier to match the pre-peak curvature of the experimental curve (because its parameters are sensitive to the nonlinear shape), so it is more direct for curve fitting. But its parameters mainly reflect a statistical description, with a physical meaning closer to “heterogeneity description”, and its link to a clear “dissipation–damage” quantitative chain is more indirect. In contrast, the energy-density-driven form has an internal variable with a clear physical direction, and it naturally connects to the Stage II energy-recursive framework. This helps keep “cyclic accumulation—final monotonic failure” consistent under one energy-driven logic. The trade-off is that it relies more on the completeness and stability of test data for energy calculation and parameter identification.

### 5.4. Effect of the test path on model evaluation

In this paper, triaxial tests were carried out with a constant confining pressure of 10MPa. They do not constitute independent validation or establish predictive accuracy for other specimens or loading paths. The short stepped program also does not establish a conventional fatigue threshold or long-term fatigue life. There is one complete valid record per temperature, with no valid replicate statistics or held-out specimens, temperatures or confining-pressure conditions. The present model was calibrated and evaluated using the same experimental dataset; therefore, the current results demonstrate consistency with the observed responses rather than independent predictive validation. Independent verification using additional specimens, temperatures or confining-pressure conditions remains necessary.

Quantitative model evaluation is limited to the measured response up to peak stress. Fracture and rapid system unloading after the peak left sparse post-peak records, which do not support quantitative characterization of complete post-peak softening or residual load-carrying behavior. Stress control does not impose a constant strain rate: changes in compliance and deformation amplitude can alter the strain-rate history. A possible rate contribution to the measured energy changes cannot be separated or quantified from the presented comparisons. Likewise, the macroscopic measurements do not isolate the contributions of heating, holding and cooling to thermal degradation. The separable damage coupling omits explicit thermal–cyclic interactions; its sensitivity to alternative coupling forms remains unassessed.

## 6. Conclusions

Based on the high-temperature heat-treatment tests and multi-level cyclic loading tests, we obtained several key findings for red sandstone under coupled conditions:

(1) The measured response depends on thermal treatment and cyclic stress level. Within a given stress level, the reported loops narrow and the additional irreversible deformation decreases over the observed cycles. Raising the stress level renews deformation and dissipation, particularly in the early cycles at the new level. These are macroscopic observations; the curves alone do not establish the respective contributions of microcracking, pore changes or mineral alterations.
(2) The reported energy results show increased input and dissipated energy at stress-level transitions and declining dissipation during subsequent cycles at the same level, with a larger recoverable share. These changes describe the tested stepped loading program. They are not evidence of a strict fatigue threshold or a general instability criterion.
(3) The staged constitutive framework combines initial compaction, history-dependent cyclic response and post-cycling monotonic loading. Its energy-driven damage evolution is phenomenological, and its thermal–mechanical coupling is a separable scalar approximation. The Weibull and energy-driven Stage III forms provide alternative descriptions fitted to the present curves. These comparisons illustrate curve reproduction; independent validation and quantified predictive performance remain to be established. Stage II uses measured residual-strain states and an inherited load-carrying state, so the current evaluation is conditional on those experimental inputs.
(4) Quantitative analysis ends at the measured peak stress. Fracture and rapid system unloading left insufficient post-peak records for reliable quantitative characterization. Additional measurements would be needed to constrain post-peak softening and residual load-carrying behavior.
(5) Future structural measurements, for example acoustic emission or CT imaging, could test the pore/crack interpretations of the macroscopic response.
(6) Further work could examine how frictional and crack-related processes contribute to dissipation and how the characteristic energy parameters depend on temperature and loading history.
(7) Application to long-term behavior, variable confinement or coupled seepage and chemical processes requires additional data and validation beyond the present tests. With one complete valid record per temperature and no valid replicate statistics, the reported trends describe these individual specimens; their variability across specimens remains unquantified.

## Supporting information

S1 Data X. (XLSX)
